# Integrated Population Modeling of Black Bears in Minnesota: Implications for Monitoring and Management

**DOI:** 10.1371/journal.pone.0012114

**Published:** 2010-08-12

**Authors:** John R. Fieberg, Kyle W. Shertzer, Paul B. Conn, Karen V. Noyce, David L. Garshelis

**Affiliations:** 1 Department of Fisheries, Wildlife, and Conservation Biology, University of Minnesota, St. Paul, Minnesota, United States of America; 2 Biometrics Unit, Minnesota Department of Natural Resources, Forest Lake, Minnesota, United States of America; 3 Southeast Fisheries Science Center, National Marine Fisheries Service, Beaufort, North Carolina, United States of America; 4 Forest Wildlife Populations and Research Group, Minnesota Department of Natural Resources, Grand Rapids, Minnesota, United States of America; University of California, United States of America

## Abstract

**Background:**

Wildlife populations are difficult to monitor directly because of costs and logistical challenges associated with collecting informative abundance data from live animals. By contrast, data on harvested individuals (e.g., age and sex) are often readily available. Increasingly, integrated population models are used for natural resource management because they synthesize various relevant data into a single analysis.

**Methodology/Principal Findings:**

We investigated the performance of integrated population models applied to black bears (*Ursus americanus*) in Minnesota, USA. Models were constructed using sex-specific age-at-harvest matrices (1980–2008), data on hunting effort and natural food supplies (which affects hunting success), and statewide mark–recapture estimates of abundance (1991, 1997, 2002). We compared this approach to Downing reconstruction, a commonly used population monitoring method that utilizes only age-at-harvest data. We first conducted a large-scale simulation study, in which our integrated models provided more accurate estimates of population trends than did Downing reconstruction. Estimates of trends were robust to various forms of model misspecification, including incorrectly specified cub and yearling survival parameters, age-related reporting biases in harvest data, and unmodeled temporal variability in survival and harvest rates. When applied to actual data on Minnesota black bears, the model predicted that harvest rates were negatively correlated with food availability and positively correlated with hunting effort, consistent with independent telemetry data. With no direct data on fertility, the model also correctly predicted 2-point cycles in cub production. Model-derived estimates of abundance for the most recent years provided a reasonable match to an empirical population estimate obtained after modeling efforts were completed.

**Conclusions/Significance:**

Integrated population modeling provided a reasonable framework for synthesizing age-at-harvest data, periodic large-scale abundance estimates, and measured covariates thought to affect harvest rates of black bears in Minnesota. Collection and analysis of these data appear to form the basis of a robust and viable population monitoring program.

## Introduction

Age-at-harvest data are commonly collected for many wildlife populations, including those of ungulates and carnivores, and a variety of methods have been developed to assess population abundances and trends from these data [1]. Recently, several authors have suggested applying modern statistical age-at-harvest models to monitor population trends, either in concert or as an alternative to more labor intensive survey methods [1]–[7]. Methods for analyzing age-at-harvest data have largely been adapted from fisheries' statistical catch-age models [8]–[9], which fall under the broader classification of integrated population (or “hidden process”) models [10]–[13]. Integrated population models synthesize demography with multiple sources of data, such as age- and length-frequencies, abundance indices, annual harvest, and life-history information, into a comprehensive analysis. A key issue with these models is that age-at-harvest data alone are insufficient to estimate population parameters (such as abundance, survival, and recruitment), in part because not all animal deaths are accounted for through hunter harvest; strong assumptions or auxiliary data are typically needed to eliminate parameters or to make them estimable [2]. Thus, a relevant question of practicality is, “what assumptions or auxiliary data are necessary to reliably estimate population trends with age-at-harvest data?”

Auxiliary datasets previously considered have included those generated by radio telemetry studies to inform survival probabilities [1], [2] and mark-recovery studies to inform survival and harvest rates [6]. Several authors have used hunter effort data to estimate harvest rates [4], [5], [14], and independent indices of abundance have been used to provide additional structure on changes in population size [5]. Laake [14] stressed that hunter effort needs to have sufficient temporal contrast to be useful for estimating model parameters, if it is the sole source of auxiliary data.

In Minnesota (MN), USA, abundance and population trends of black bears (*Ursus americanus*) are monitored to inform each year's allocation of hunting licenses. Whereas trend information has been gleaned from a variety of indices, including harvest data, periodic statewide mark–recapture estimates have provided the primary tool for assessing the status of the population. Marking occurs via ingested tetracycline-laced baits spread across the bear range, and recaptures consist of bone samples (examined for tetracycline marks) submitted by hunters [15]. These estimates are labor and cost intensive, and therefore have been conducted infrequently (∼5–6 year intervals). Moreover, uncertainty associated with each yearly estimate has hampered assessment of population trend [16]. As such, we were interested in determining whether age-at-harvest modeling could aid or supplant statewide population estimates as a means of estimating changes in bear abundance.

We built plausible models of MN black bear population and harvest dynamics by integrating age-at-harvest data with statewide mark–recapture estimates of abundance available for 1991, 1997, and 2002. In addition, we used data from a long-term (nearly 30 year) radio-telemetry study (Appendix S1) to provide guidance on the form of the model (e.g., structure of harvest rates), but not to estimate model parameters. Although these additional data could, in principle, have been integrated into the model fitting process, we chose to reserve these data for the purposes of testing the validity of model outputs. We had two primary research objectives. First, we aimed to evaluate the robustness of these models using a large-scale simulation study. Although integrated population models are gaining momentum in wildlife studies, surprisingly little testing has been conducted on their behavior (but see [13], [17]). Second, guided by the simulation study, we applied the models to actual data with the intent of informing natural resource management.

## Methods

### Model Development

Our application is based loosely on the “stock synthesis” framework [18], [19], which has been used widely in fishery stock assessments since the early 1990s. The model is conditioned on initial (estimated) abundance at age, and then it projects the age-structured population forward through time, fitting to available data. The dynamics of the population are determined by estimated births (cubs) and mortality, including harvest.

**Figure 1:**
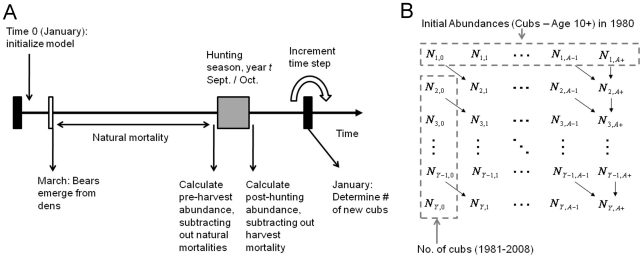
A visual depiction of the timeline used in black bear population models. A number of processes (including non-hunting mortality, harvests, and recruitment) govern annual changes in abundance and age composition (Panel A). These processes are summarized in a Markovian model for population dynamics (Panel B) where abundance parameters (*N_t,a_*) pertain to the number of animals in age class *a* in January of year *t*. Model fitting is accomplished by minimizing the difference between observed and expected bear harvests via a χ^2^ objective function.

Using the notation summarized in Table 1, and a timeline guided by knowledge of life history of black bears in MN (Figure 1A), annual changes in abundance are given by the following set of equations:
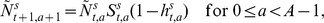
(1a)

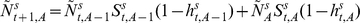
(1b)These quantities are related to observed data through predictions of age-at-harvest records each year, where
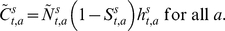
(2)


**Table 1:** Parameters, functions of parameters, and statistics used in the age-at-harvest model and auxiliary analyses for Minnesota black bears.

Parameter or function of parameters	Description
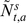	Predicted abundance of age *a*, sex *s* individuals in year *t* ( 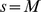 denotes males; 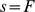 denotes females) by the statistical age-at-harvest model.
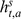	Finite harvest probability of age *a*, sex *s* individuals in year *t*
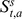	Finite probability of surviving all non-hunting sources of mortality for age *a*, sex *s* individuals in year *t*
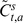	Predicted harvest of age *a*, sex *s* individuals in year *t* by the statistical age-at-harvest model. This quantity is given by 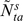 (1− 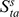 ) 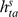 .
Statistics	
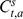	Number of age *a*, sex *s* individuals harvested in year *t* (observed age-at-harvest matrix, corrected for sex misclassification rates, and inflated to account for bears that were not aged).
*A*	Number of ages included in the population model. Once an individual reaches age class *A*, they are assumed to stay in that age class until they die (i.e., it is treated as a ‘plus’ group, and thus, labeled as *A* ^+^).
*T*	Number of years age-at-harvest data are available

This formulation assumes no mortality between the end of the hunting season and the time at which new individuals are recruited into the population (Figure 1A). This is a realistic assumption for MN black bears, which enter winter dens (where mortality is nearly zero) during or immediately after the September–October hunting season, and give birth in January.

The model as articulated thus far is overparameterized; that is, it is impossible to estimate all parameters with an age-at-harvest dataset alone. Thus, we made several simplifying assumptions to reduce the dimensionality of the problem. We assumed 50% of cubs were male and set survival rates of cubs to 0.76 for males and 0.88 for females, values estimated from den checks of radio-collared adult bears near the center of MN's bear range (Appendix S1). Because bears are not legally hunted until 1.5 years old, parameters for cub and yearling survival from den emergence to the fall hunting season at age 1.5 are confounded with fecundity parameters (number of cubs produced per year). Thus, we also fixed yearling survival of males (at a value of 0.88), but estimated a parameter that reflected the difference between male and female annual survival, constrained to provide the biological realism of higher female survival. In addition, we assumed non-hunting mortality rates were constant with respect to age and time for all bears age 2 or older. Thus, our models captured non-hunting mortality using three estimated parameters (one each for females age 1, males ages 2+, and females ages 2+).

We considered two different sub-models for harvest rates, parameterized on a complementary log-log scale, and applied to an age-at-harvest data matrix consisting of 29 years (1980–2008) and 10 age classes (ages 1–9, 10+). In the first configuration, we modeled temporal variation in harvest rates by regressing on an index of natural food availability and an index of hunting effort (the number of estimated bear hunters; [20]); additional harvest vulnerability parameters were estimated in the first four years since these covariates were not available for 1980–1983. In this approach, a total of seven regression parameters were used to account for temporal variability in harvest probabilities (Box S1). In the second configuration, we estimated annual fluctuations in harvest vulnerabilities using an unstructured model. This approach required 29 parameters (1 for each year) to account for temporal variability. With both approaches, we modeled a nonlinear effect of age using natural cubic regression splines with 3 degrees of freedom (interior knots were set at ages 2 and 7, and outer knots were set at ages 1 and 10), and we included an extra parameter to account for sex differences. Age, sex, and temporal effects were assumed to be additive on the log-log scale. We created the regression spline basis functions using the ‘ns’ function in R [21], [22]. We chose age = 2 as an interior knot to allow greater flexibility to fit early ages and age = 7 to allow for an inflection point shortly after maturity. We refer to these two different harvest sub-models as *H*(*a*, *s*, *f*, *e*) and *H*(*a*, *s*, *yr*), where the subscripts refer to age (*a*), sex (*s*), food availability (*f*), hunting effort (*e*) and individual year effects that were unstructured by food or hunting effort (*yr*).

### Model Fitting

Model fitting was accomplished by minimizing the difference between observed and predicted harvest at age via a χ^2^ objective function [23],
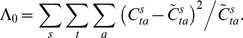
(3)The χ^2^ objective function was appealing because its evaluation only required specification of the population dynamics model to project counts and estimate harvests through time, rather than a large set of distributional assumptions meant to reflect both sampling and process variability. In addition, it avoided potential numerical problems associated with large combinatoric terms in product binomial or multinomial likelihoods sometimes used for fitting age-at-harvest models. Lastly, in limited initial testing, we found the optimizer was also more likely to converge to a minimum (as indicated by a positive definite Hessian matrix) when using a χ^2^ objective function compared to least squares. Assuming the population dynamic model gives a reasonable approximation to reality, Λ_0_ should converge to a 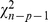 distribution, where *n* = 580 is the number of unique cells (2 sexes×29 years×10 age classes) and *p* is the number of estimated model parameters.

In addition to fitting age-at-harvest data, we explored the usefulness of incorporating independent abundance estimates from statewide, tetracycline mark-recapture studies conducted in 1991, 1997, and 2002 [15], [16]. To accomplish this, we added a penalty term to the χ^2^ objective function, minimizing
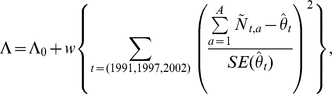
(4)where 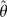 and SE(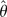) represent the point estimate and standard error associated with the mark-recapture study in year *t* [16] and 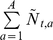 is the model-based estimate of abundance in year *t*, excluding cubs (which were not part of the mark-recapture study). Because 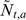 is a function of model parameters, this compound objective function provides a means to tune parameters to both harvest data and independent mark-recapture abundance estimates, with *w* determining the relative weights given to these two sets of information.

### Application to MN black bears

MN hunters were required to register harvested bears and report the sex. For the purposes of modeling, we used registered harvest data for 1980–2008, except that we corrected these data for mis-reported sex using data from harvested radio-collared bears of known sex. Of 159 harvested radio-collared female bears, 17 were misclassified as males, whereas only 1 of 183 harvested males was misclassified as female.

Age-at-death data for harvested bears were obtained from teeth submitted by hunters. Ages were estimated by decalcifying, cutting and staining tooth samples, and then counting annuli under a microscope. Tooth submission was initially voluntary, but made mandatory in 1986; compliance, however, was imperfect, and some submitted teeth were broken. Usable teeth averaged 71% of the annual harvest. We inflated age-at-harvest records accordingly, assuming that the distribution of ages from submitted teeth represented the overall harvest. We also assumed that age interpretations from teeth were made without error. Potential errors in age interpretations were partially alleviated by grouping all ages >9 years old into a single category (10+), as teeth with many annuli are the most difficult to count. Although each year some cubs were harvested, this was not legal, and cubs were eliminated from the dataset.

We fit both harvest sub-models to our MN black bear data and integrated the mark-recapture abundance estimates using three different penalty weights, *w* = (0, 1, or 200) in eq. 4; note, *w* = 0 indicates the mark-recapture estimates were not used in the model fitting process. The largest value was chosen because it put the two sets of information (age-at-harvest and mark-recapture data) roughly on the same scale. We refer to these six estimators as *H*(*a*, *s*, *f*, *e*; *w* = 0, 1, or 200) and *H*(*a*, *s*, *yr*; *w* = 0, 1, or 200).

We used a bootstrap approach to explore parameter uncertainty. For each of 1000 bootstrap replicates, we: 1) resampled the dataset of harvested bears with known ages to form a new observed age-at-harvest matrix; 2) resampled the telemetry data set used to estimate sex misclassification rates; 3) applied bootstrap estimates of correction factors (from step 2) to the data formed in step 1 and then inflated the resulting sex-specific age-at-harvest matrices for the percentage of harvested bears that were aged in each year; and 4) used a parametric bootstrap (sampling normal random deviates) to generate new mark-recapture estimates in 1991, 1997, and 2002. We then applied each of the 6 estimators to the bootstrap data sets and summarized the output using percentile based intervals. All models were fit using AD Model Builder software [24], and we utilized ADMB2R [25] to facilitate post-processing in R. We provide sample AD Model Builder code in an accompanying online supplement (Appendix S4).

### Simulation Study

We conducted a set of eight simulation experiments to test robustness of the overall modeling approach, to narrow the list of candidate models, and to better understand model results (see Appendix S2 for a detailed description of the simulation study). In each case, we tested the models using the same basic process: 1) starting with an initial population structured by age and sex, we applied an operating model that described population and harvest dynamics; 2) time series of abundance and harvest were sampled to generate data available for building integrated population models; and 3) integrated population (estimation) models were applied to the simulated data to determine if characteristics of the operating model (e.g., abundance trends) could be recovered. These simulations included scenarios in which the operating and estimation models differed.

**Figure 2:**
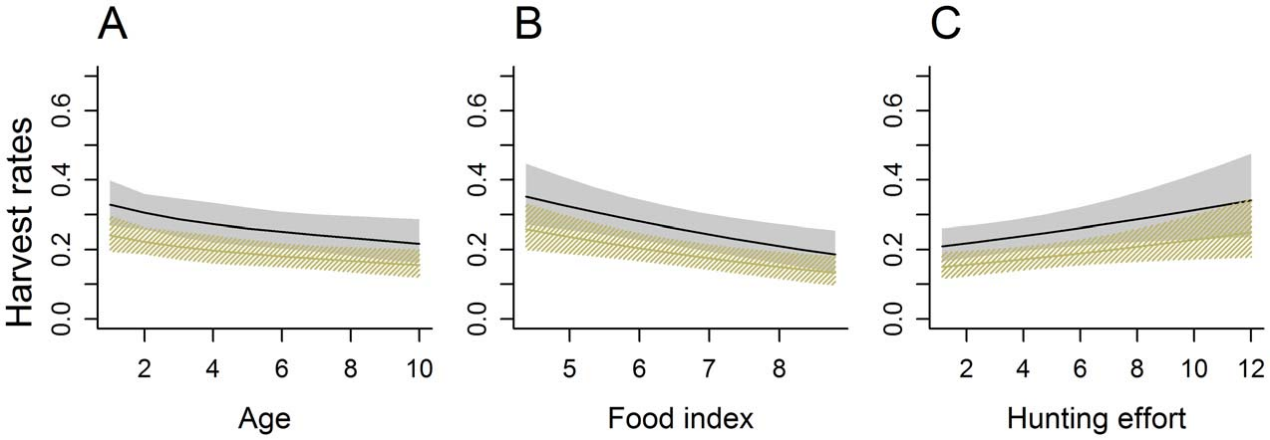
Estimates of harvest rates from telemetry data. Harvest rates for males (black solid lines) and females (tan lines) were estimated by fitting the *H*(*a*, *s*, *f*, *e*) sub-model to telemetry data collected from 1982–2004 and regional estimates of food availability and hunting effort (Appendix S1). Data were collected from the central part of the state of Minnesota. In each panel, covariates not displayed on the *x*-axis were held constant at values of age = 5, food index = 6.5, and hunting effort 6.5. Shaded areas represent pointwise 90% confidence intervals constructed using asymptotic likelihood methods (standard errors were calculating using the inverse of the Hessian matrix, and confidence limits were calculated on the scale of the linear predictor and then transformed to the nominal [harvest rate] scale).

We began with a “Baseline” simulation scenario using the *H*(*a*, *s*, *f*, *e*) sub-model to capture temporal variability in harvest rates. Specifically, we set harvest regression parameters to values estimated from analyzing harvest mortality of radio-collared bears (Figure 2; Appendix S1). These data indicated that harvest rates varied temporally as a function of food availability and hunter effort, and also varied nonlinearly with age. We also set survival rates for cubs (0.76 for males, 0.88 for females), male yearlings (0.88), and the sex ratio at birth (50∶50) in the estimation model to values applied in the operating model. Demographic stochasticity in harvest and survival was simulated using binomial random variates in each of the 29 simulated years (1980–2008), but otherwise, the projections were deterministic (e.g., the number of cubs in each year was constant across simulations). The model resulted in a mean abundance trajectory that steadily increased during the first 15 years (1980–1995) before leveling off for the remainder of the time series, similar to trends suggested by the mark-recapture data.

Six other scenarios were constructed by altering, one at a time, single facets of this “Baseline” scenario: temporal variability in non-hunting mortality and harvest rates (labeled “Stochastic Rates”), temporal trends in harvest rates (“Trends in Harvest”), temporal trends in survival probabilities (“Increasing *S*(*t*)”), underreporting of yearling bears (“Reporting Error”), incorrect assumptions regarding cub and yearling survival (“Incorrect Survival”), or an interaction effect on harvest between natural food availability and sex (“Food×Sex Effect”). A final scenario (“Kitchen Sink”) was constructed by including all of those deviations simultaneously.

Additionally, we estimated trends using the Downing population reconstruction method [26], a procedure that others have tested and employed on bears and other game mammals [3]. Downing's method is a conceptually simpler approach that “reconstructs” the population from total harvest and age-at-harvest data, using calculations that can be implemented easily with common spreadsheet software. It assumes a constant ratio of natural to hunting mortality, and ignores the former in the reconstruction, thus, providing an estimate of abundance for the portion of the population that is ultimately killed by hunters. Davis et al. [3] used extensive computer simulations to test this method, and concluded that it provides a robust approach to trend (but not total abundance) estimation, even with collapsed older age classes, which is necessary to estimate trends for recent years. Thus, the Downing method may be useful as a simple alternative to more complex integrated age-at-harvest models for population monitoring. In our simulations, we applied the Downing method with older ages collapsed to 3+ years old (as recommended by [3]) for males only, females only, and males and females combined.

For each scenario, we simulated 1000 datasets and applied each of the six estimators (two harvest configurations×three penalty weights [*w* = 0, 1, or 200]), as well as the three Downing reconstructions (males, females, or both). For the estimation approaches that included mark-recapture data, we generated abundance estimates in 1991, 1997, and 2002 as random normal deviates with mean set equal to the true population size and standard deviations set to achieve a CV of 8% (the average CV observed in our mark-recapture studies); in all cases, we set 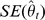 to the value that resulted in an 8% CV.

### Evaluation of Simulation Results

During initial simulation testing, we observed cases where estimates of abundance were scaled too high or too low, yet exhibited similar trends as the true abundance time series generated by the operating model. Because trends in abundance are often useful for management, we desired performance metrics that would allow us to separately evaluate 1) the model's ability to return the correct overall abundance scale, and 2) the model's ability to accurately portray changes in abundance. To evaluate (1), we compared the true mean abundance (averaged across years and simulation runs) to the average estimated abundance (again, averaged across years and simulation runs). To evaluate (2) we compared true and estimated yearly transitions, λ*_t_* = *N_t_*_+1_/*N_t_*, as well as the mean squared error (MSE) of estimated transitions (again, over years and simulations). For the purposes of this paper, we define MSE(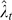) = 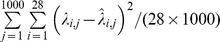; however for reporting MSE, we multiplied values by 1000.

## Results

### Simulation Experiments

We highlight the main results of the simulation study here, but refer the reader to Appendix S2, Table S1, and Figure S1 for a more detailed summary, including performance statistics for all six age-at-harvest model estimators and three Downing reconstruction estimators applied to each simulation scenario. Age-at-harvest models converged to values that minimized the objective function (as indicated by positive definite Hessian matrices output from AD Model Builder) in >96% of the simulations for each scenario. When averaged across time steps (and simulations), the abundance estimator *H*(*a*, *s*, *f*, *e*; *w* = 0) was biased high in seven of the eight scenarios (the exception was Food×Sex Interaction scenario in which it was biased low), whereas the *H*(*a*, *s*, *yr*; *w* = 0) estimator was biased high in five scenarios and biased low in three scenarios (Table S1). However, the bias was often small. For example, in the Baseline Scenario the true mean abundance (in thousands) was 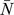 = 13.96 compared to 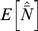 = 14.09 and 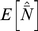 = 15.33 for the *H*(*a*, *s*, *f*, *e*; *w* = 0) and *H*(*a*, *s*, *yr*; *w* = 0) estimators, respectively (Table S1). Incorporating the mark-recapture data into the objective function always resulted in less biased estimators of mean abundance, and in some cases, no bias at all (Table S1).

**Figure 3:**
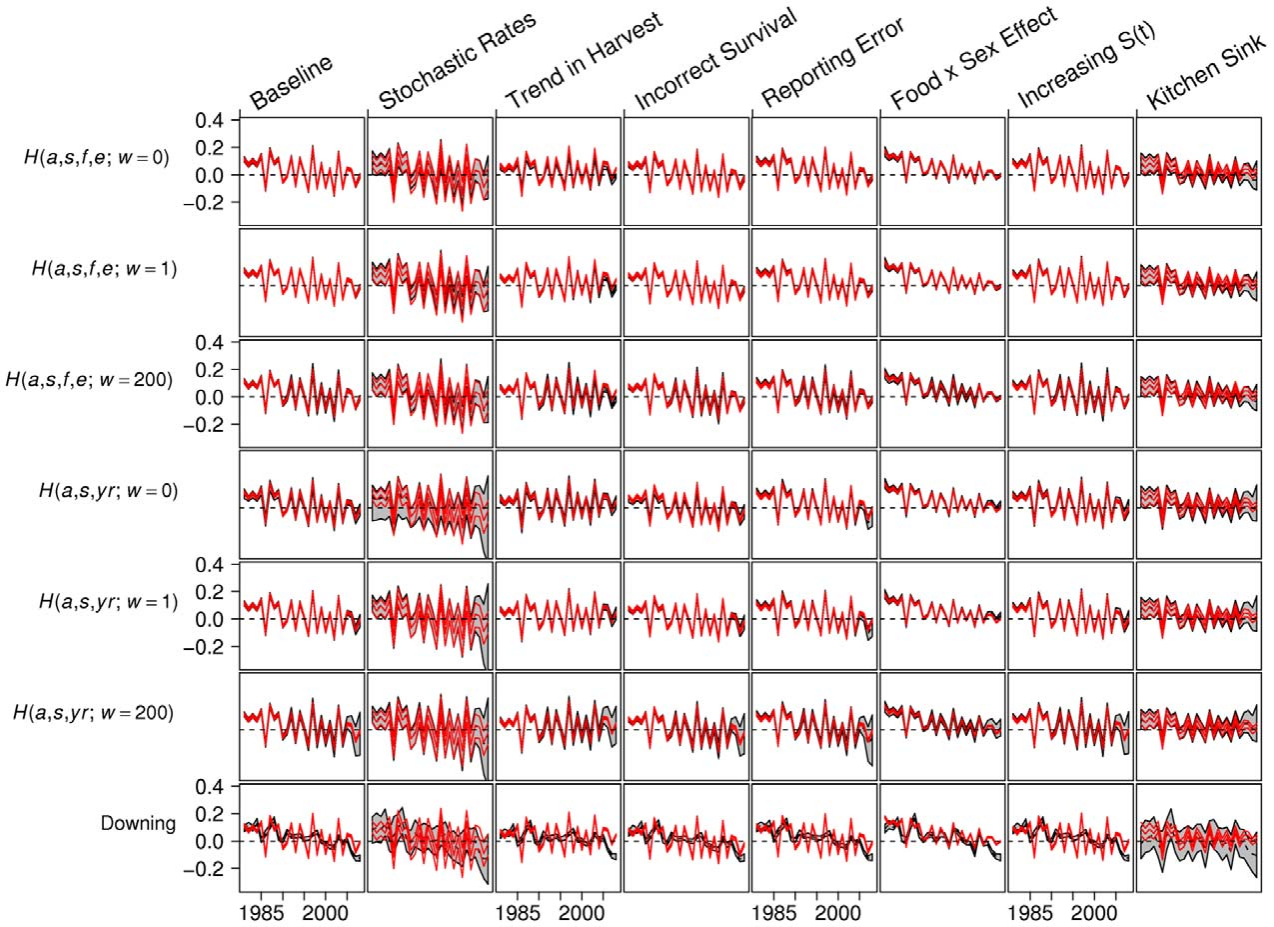
Performance of yearly trend estimators with simulated data. In each panel, the y-axis depicts values of log(λ*_t_*), where λ*_t_* = *N_t+1_/N_t_*. Red lines correspond to (2.5^th^, 50^th^, and 97.5^th^ percentiles) of the true population dynamics (across stochastic simulations). Gray polygon encompasses 95% of the estimated values. Rows correspond to different estimators (see Box S1) and columns correspond to different simulation scenarios. In all plots, the horizontal dashed black line corresponds to log(λ*_t_*) = 0.

Although the abundance estimators were biased in several of the simulation scenarios, estimates of annual trends were remarkably accurate and robust to model mis-specification (Figure 3). For most scenarios and estimators, the distribution of 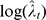 values over time was similar to the distribution of true log(λ*_t_*) values. One exception was the *H*(*a*, *s*, *yr*; *w* = 0) estimator in the Stochastic Rates scenario, where the model was occasionally unstable, resulting in large initial abundances and negative values of log(λ*_t_*) (Figure S1). In addition, trend estimates for the last 4–5 years of the time series were highly variable for the *H*(*a*, *s*, *yr*) sub-models in the Stochastic Rates and Kitchen Sink scenarios (Figure 3).

MSE(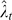) was nearly always smaller when mark-recapture estimates were included without weighting (*w* = 1) compared to estimators that weighted this component of the objective function (*w* = 200), and this held true for both harvest model parameterizations (Table S1). In six of the eight scenarios, the *H*(*a*, *s*, *f*, *e*; *w* = 1) estimator resulted in the smallest MSE(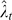). In the other two scenarios, Trend in Harvest and Kitchen Sink, the *H*(*a*, *s*, *yr*; *w* = 1) resulted in the smallest MSE(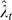). In these latter two scenarios, the *H*(*a*, *s*, *f*, *e*) sub-models underestimated harvest rates at the start of the time series and overestimated harvest rates at the end of the time series because these simulation scenarios included a systematic trend in harvest rates not attributable to changes in food availability or hunting effort (Appendix S2). As a result, these models overestimated abundance at the start of the time series and underestimated abundance at the end of the time series (Figure S1). By contrast, the *H*(*a*, *s*, *yr*) sub-models estimated a separate harvest vulnerability in each year, and were thus not impacted by trending harvest rates.

MSEs of annual changes in abundance from all of the age-at-harvest models were an order of magnitude smaller than those derived from Downing's reconstruction method (Table S1). Although the Downing method largely captured the long-term trend in the population (increasing trajectory at the start of the time series, fairly stable trajectory at the end of the time series), collapsing age classes smoothed over the true trajectory, resulting in yearly transition estimates that were often out-of-phase with the true annual growth rates (λ*_t_*) (Figure 3, bottom row).

### Application to MN black bears

**Figure 4:**
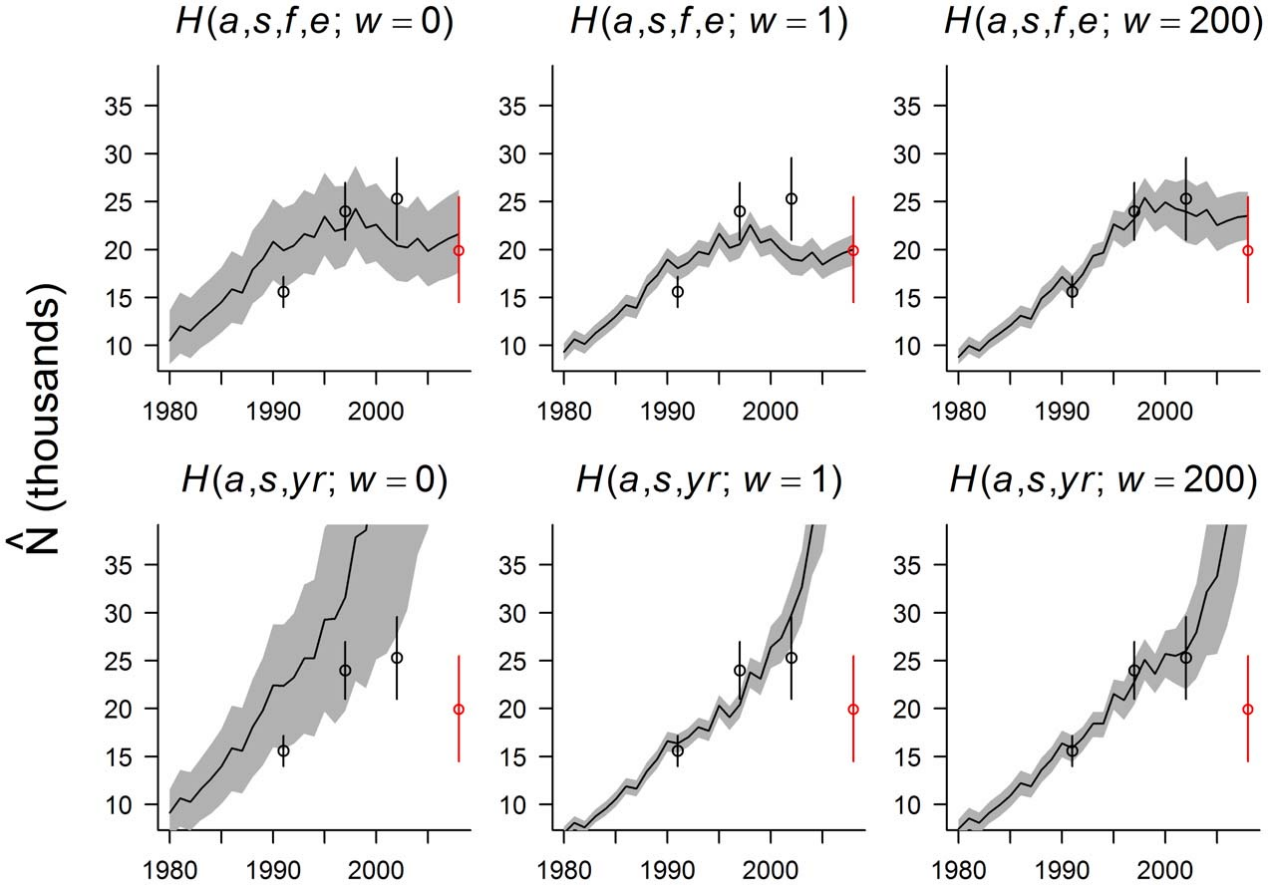
Abundance estimates from models fit to Minnesota black bear data (1980–2008). Circles with vertical lines depict independent mark-recapture estimates (and corresponding 95% CIs) in years (1991, 1997, 2002, 2008). *H*(*a*, *s*, *f*, *e*) sub-models account for temporal variability in harvest rates as a function of food availability and hunting effort indices, whereas the *H*(*a*, *s*, *f*, *yr*) sub-models use an unstructured model for harvest rates. In both cases, *w* refers to the weight assigned to the mark-recapture component of the objective function used to fit the model (note: the 2008 mark-recapture estimate, colored in red, was not used in the model fitting process). Shaded areas in each panel depict pointwise 95% variability bands estimated using a bootstrap with 1000 replicates.

The *H*(*a*, *s*, *f*, *e*) sub-models, when fit to MN black bear data, estimated an initially increasing abundance trend, followed by a leveling off around 1995, whereas the *H*(*a*, *s*, *yr*) sub-models all resulted in rapidly increasing populations at the end of the time series (Figure 4). The recent increase in the *H*(*a*, *s*, *yr*) sub-models coincided with estimates of decreasing harvest rates (Appendix S3). Similar to the simulation study, bootstrap intervals for both harvest sub-models suggested estimators with *w* = 1 were least variable, followed by those with *w* = 200, then *w* = 0.

**Figure 5:**
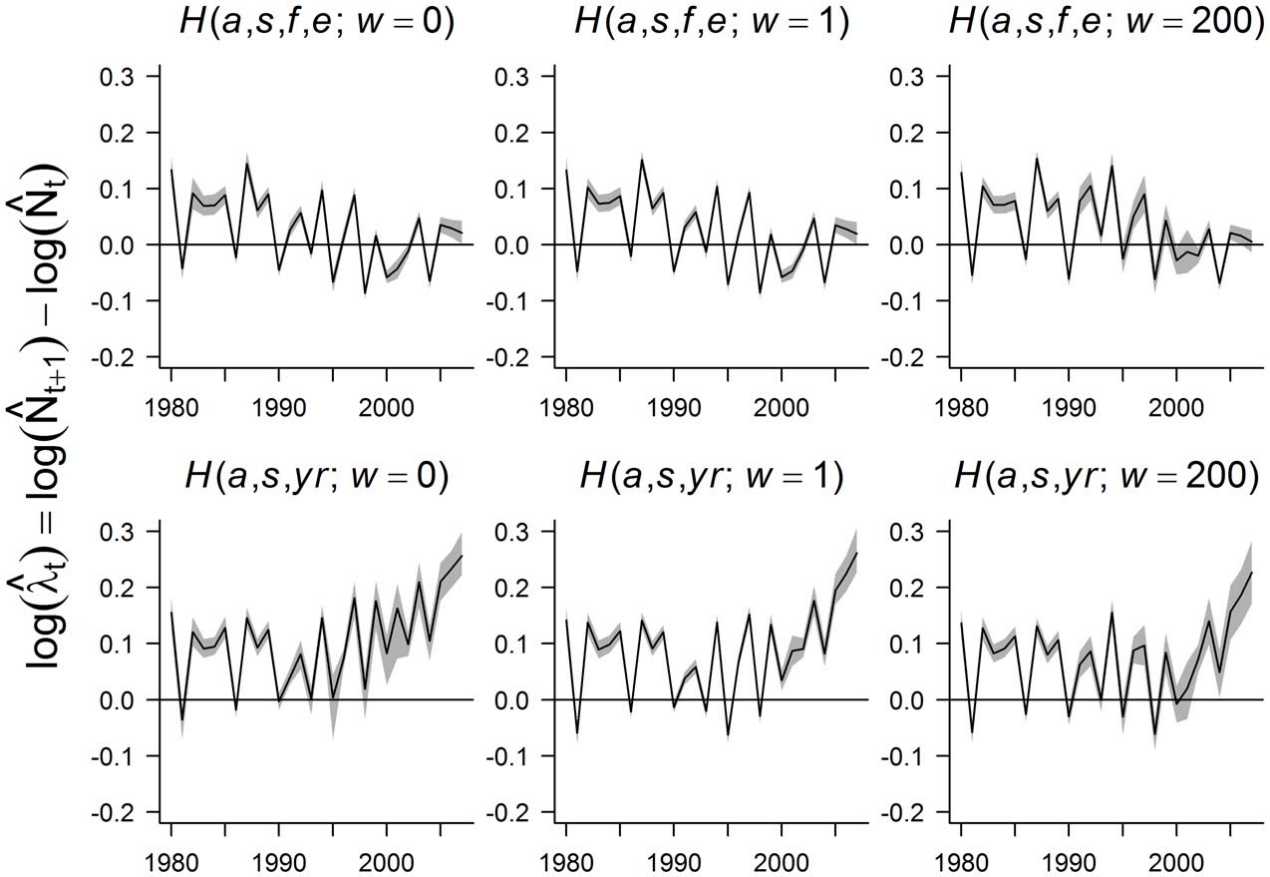
Estimated annual trends in abundance from models fit to Minnesota black bear data (1980–2008). Annual trends are given by log(λ*_t_*) = log(*N_t_*_+1_)−log(*N_t_*). The horizontal line at log(λ_t_) = 0 corresponds to a stable population. *H*(*a*, *s*, *f*, *e*) sub-models model temporal variability in harvest rates as a function of food availability and hunting effort indices, whereas the *H*(*a*, *s*, *yr*) sub-models use an unstructured model for harvest rates. In both cases, *w* refers to the weight assigned to the mark-recapture component of the objective function used to fit the model. Shaded areas in each panel depict pointwise 95% variability bands estimated using a bootstrap with 1000 replicates.

Given the relative instability of the *H*(*a*, *s*, *yr*) sub-models in the Stochastic Rates scenario and the biologically unrealistic trends and abundance estimates obtained from fitting these models to the MN black bear data, we limit subsequent focus to the *H*(*a*, *s*, *f*, *e*) sub-models. The *H*(*a*, *s*, *f*, *e*; *w* = 0) estimated abundance was significantly higher than the corresponding mark-recapture estimate in 1991, but model-based estimates passed through the 95% confidence intervals for the 1997 and 2002 mark-recapture estimates (Figure 4). Models with *w*>0 will be penalized most heavily for not fitting the 1991 mark-recapture estimate, since this estimate had an associated SE that was considerably lower than those in 1997 and 2002. Correspondingly, the *H*(*a*, *s*, *f*, *e*; *w* = 1) abundances were lower than those of the *H*(*a*, *s*, *f*, *e*; *w* = 0) estimator. Yet, they remained above the 1991 mark-recapture estimate and also passed below the 2002 mark-recapture estimate. The *H*(*a*, *s*, *f*, *e*; *w* = 200) population trajectory passed through the confidence intervals associated with all three mark-recapture estimates. Although the estimated abundance trajectories for the different *H*(*a*, *s*, *f*, *e*) sub-models appeared to differ substantially, population growth trends depicted by log(λ_t_) = log(*N_t_*_+1_)−log(*N_t_*) were largely similar (e.g., the models agreed on the sign of log(λ_t_) in all but 1 year, 1993; Figure 5).

**Figure 6:**
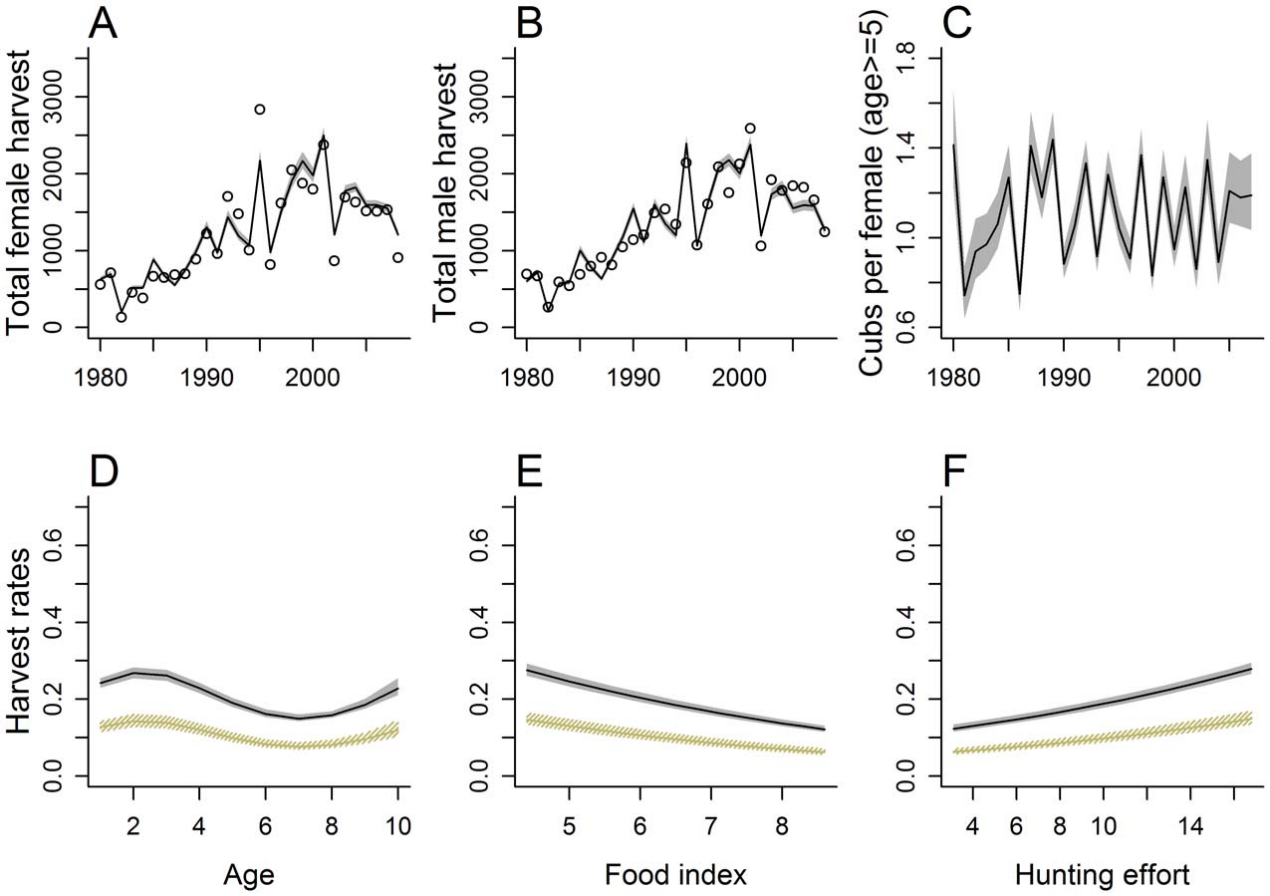
Model-based predictions from the *H*(*a*, *s*, *f*, *e*; *w* = 1) estimator applied to Minnesota black bear age-at-harvest data. Panels A and B give model based estimates of total female and male harvests (lines) along with the empirical data (points). Panel C gives model based estimates of the number of cubs per female >age 5 over time. Panels D–F give model based estimates of harvest rates for males (black lines) and females (tan lines) as a function of age, food availability, and hunting effort (covariates not displayed on the *x*-axis were held constant at values of age = 5, food index = 6.35, and hunting effort = 10.21). Shaded areas in each panel depict pointwise 95% variability bands estimated using a bootstrap with 1000 replicates.

Estimates of harvest rates (as a function of age, sex, food availability, and hunting effort) were similar for the three *H*(*a*, *s*, *f*, *e*) sub-models (Appendix S3), so we will subsequently only focus on the *H*(*a*, *s*, *f*, *e*; *w* = 1) estimator, which provided a good overall fit to the harvest data (Figure 6A,B).

Estimates of harvest rates increased with decreasing levels of natural foods and increasing hunting effort (Figure 6E,F), similar to relationships estimated from telemetry data (Figure 2B,C). On the other hand, the fitted age-at-harvest models resulted in a highly non-linear relationship as a function of age, with harvest rates of 2-year-old bears higher than those of yearlings, a decrease in harvest rates from age 2 through age 7, and then increasing harvest rates from age 7 to age 10+ (Figure 6D). By contrast, estimates from radio-telemetry suggested that harvest rates decreased nearly linearly with age (Figure 2A).

The estimated number of cubs divided by the estimated number of females (≥age 5), a model-based estimate of recruitment strength, exhibited 2-point cycles (Figure 6C). This pattern occurs because females tend to produce cubs every two years, and in MN (and elsewhere) they become somewhat synchronized by widespread food failures when many fail to produce but then produce the following year [27]. That these cycles resulted, without directly fitting data on cub production, validates the models' ability to capture real biological phenomena. Such validation should be reassuring to wildlife managers.

## Discussion

Animal populations are notoriously difficult to monitor because of costs and logistical challenges associated with collecting informative data on population trends and abundance. Mark-recapture studies are particularly difficult to apply on large geographical scales, and provide only a snapshot of abundance. Black bears pose their own set of problems, as ear tag loss [28] and behavioral responses to baiting [16] may bias estimators. Further, repeated surveys are necessary to yield information on population trend, which still may be equivocal due to estimation errors or an inadequate time series of population estimates [16]. Nevertheless, jurisdictions that hunt species like black bears require estimates of abundance and information on population trend to make effective adaptive management decisions. For bears, management agencies rarely rely strictly on population estimates to assess trend [29], instead often employing a loose collection of information, including indices derived from harvest data (e.g., changes in age structure, sex ratios, hunting success, bears killed per unit of hunting effort, etc.) despite known problems with these approaches [30], [31]. Many agencies collect age-at-harvest data, but have underutilized this information as a population monitoring tool. Our study has shown that these data can be highly informative of population trend, and when calibrated with actual population estimates, also provide useful estimates of abundance.

Simple deterministic population reconstruction methods, such as the Downing approach, are relatively easy to apply and may provide reasonable depictions of population trends when harvest rates and survival parameters do not vary greatly over time [1], [3]. The Downing method shares many characteristics with our integrated modeling approach (e.g., both model harvest rates as a function of age and time), and one might argue that the trends from the Downing method could be scaled by the mark-recapture estimates to provide a similar depiction of population abundance over time. Integrated population models require additional auxiliary data (or assumptions) and more sophisticated technical expertise, but they are appealing because they provide a formal framework for combining disparate data sources, and they offer the potential to estimate additional parameters of interest [1]. In our example application, age-at-harvest models enabled us to explore links between natural food availability, hunting effort, and harvest rates – relationships that will likely prove useful for management. In addition, these methods more accurately reflected annual changes in abundance than the simpler Downing reconstruction method.

In our simulation study, we found that abundance estimates were often biased, but trends were reassuringly accurate and robust to model mis-specification. Yet, the *H*(*a*, *s*, *yr*; *w* = 0) estimator occasionally performed poorly in the Stochastic Rates scenario. We suspect these latter models illustrate a common identifiability problem with age-at-harvest data: similar harvest numbers can occur from high abundance with low harvest rates or low abundance with high harvest rates. As such, estimates from such models should be viewed with caution. By contrast, the *H*(*a*, *s*, *f*, *e*) sub-models rely on considerably fewer parameters, instead using patterns in natural food availability and hunting effort to predict harvest probabilities. In essence, these models are more constrained, resulting in greater stability. Importantly, adding the mark-recapture abundance estimates to the objective function substantially improved the performance of the *H*(*a*, *s*, *yr*) sub-model in the Stochastic Rates scenario (Figure 3) and also resulted in less biased estimates of mean abundance for both sets of model estimators in all scenarios (Table S1). Thus, these additional data appear crucial for correctly estimating the scale of abundance, particularly when covariates that explain variation in harvest rates are unavailable.

Applying our models to black bears in MN, we found that the exponentially increasing population trends estimated for MN black bears from the fitted *H*(*a*, *s*, *yr*) sub-models were biologically unrealistic, with population growth rates (λ) for recent years exceeding 0.2 (Figure 5). This is not possible in a population where bears were being harvested at rates of ∼20% (estimated from harvests of radio-collared animals across the state, MNDNR unpublished data). Conversely, models including effects of food and hunter effort provided highly plausible population trajectories that not only matched trends in various population indices (e.g., hunting success, sightings, nuisance activity, all of which were stable or declining in recent years; MNDNR unpublished data), but also intersected a population estimate obtained after the modeling work was completed (2008; Figure 4).

Inclusion of mark-recapture estimates helped scale the *H*(*a*, *s*, *f*, *e*) sub-models, and also influenced estimates of population trend to a lesser extent (Figure 5). The integrated population model estimators with *w* = 1 and *w* = 200 attempt to strike a balance between fitting the harvest data and the mark-recapture point estimates, thus providing a more robust estimate of trend. Even when weighting the mark-recapture data heavily (i.e., in the *H*(*a*, *s*, *f*, *e*; *w* = 200) model), the integrated population models yield a different pattern of population change than do the series of individual mark-recapture abundance estimates. We believe these calibrated models are likely to be more useful to managers than the periodic statewide mark–recapture estimates alone, because 1) the modeled estimates yield trend information, which is difficult to glean from a limited number of population estimates, 2) the modeled estimates smooth over sampling variability and biases that can affect individual empirical estimates [16], and 3) the modeled estimates provide information for the most recent years, even if the last mark–recapture estimate was several years in the past (Figure 4).

Age-at-harvest models can and should be tailored to available data, and their performance should be evaluated in light of these data (e.g., using realistic simulation studies). This approach to model testing and evaluation can help to provide context for interpreting applied results and also suggest what data should be collected in the future to improve model performance and resulting management advice. For example, the robustness of trend estimates across several simulated scenarios with model mis-specification, particularly for the *H*(*a*, *s*, *f*, *e*; *w* = 1) and *H*(*a*, *s*, *f*, *e*; *w* = 200) estimators, helped to increase confidence in our estimates of relative abundance when these models were applied to the MN black bear data. Lastly, the biologically unrealistic predictions from the *H*(*a*, *s*, *yr*) sub-models, when fit to MN black bear data, are not surprising given the occasional instability of these models near the end of the time series in the simulation study. It is the end of the time series that is most important for management, and where the Downing model lacks predictive ability.

Although we explored the use of mark-recapture estimates of abundance in fitting models to the age-at-harvest data, we chose not to directly incorporate additional estimates of adult survival or estimates of harvest regression parameters from available telemetry data. The latter were collected in more localized study areas and might not agree well with statewide patterns. However, the close agreement between the *H*(*a*, *s*, *f*, *e*) sub-models and models fit independently to these telemetry data (i.e., the similar relationships between harvest rates and food abundance, hunter effort indices depicted in Figure 2 and Figure 6) provides further support for the age-at-harvest models. Estimates of age-related patterns agreed to a lesser extent, and we suspect any discrepancies likely result from the mis-specification of one or more parts of the model. One possibility is that the unrealistic increase in harvest rates for older bears (age>7; Figure 6D) represents the model's attempt to account for an increase in non-hunting mortality rates, which were constrained to be constant for all bears ≥2 years old. Although no simulation study can fully exhaust all possible sources of error, our testing suggests model-based estimates of abundance trends can be fairly robust to model mis-specifications. Further, the models' ability to translate signals from the data into known biological phenomena (e.g., 2-point cycles in reproduction rates) is reassuring.

One major assumption of age-at-harvest models is that the age distribution data obtained from the harvest are reasonably representative of the total harvest, as rarely would any management agency have access to age information on every harvested animal. We were missing data for 30% of the harvest, because some hunters chose not to comply with the mandatory tooth submission (given that there was no penalty for non-compliance), forgot to comply, were unable to comply (e.g., left skull with taxidermist), or tried to comply but failed (e.g., tooth broken during extraction, or lost in mailing). We originally conjectured that hunters who killed small (young) bears might have been less likely to submit a tooth sample, either fearing it was a cub (illegal to harvest), or presuming it was a yearling (and so not interested in learning the age from the tooth sectioning). Our simulation study suggested that this phenomenon would produce an under-representation of yearlings in the age data, resulting in lower estimated harvest rates for yearlings than for 2 year olds, a pattern observed in estimates from our age-at-harvest models (Figure 6D) but not our telemetry data (Figure 2A). Thus, another benefit of the simulation approach is the ability to detect deficiencies in the data, and to determine how these deficiencies might influence model estimates and predictions. In this case, our simulation modeling suggested that trends estimated from analyzing age-at-harvest data should provide robust results even with underreporting of yearlings. Aging error is also commonplace when relying on cementum annuli [32]. Conn et al. [17] simulated typical patterns of aging error from cementum annuli analysis, and found they did not have large impacts on estimates of abundance derived from age-at-harvest data.

We expect to see increased interest in applying age-at-harvest models to wildlife data in coming years, particularly given recent applications in the literature [1], [2], [4]–[6]. There are many ways to fit age-at-harvest models and to judge model reliability. We chose to fit models that included only fixed effects parameters, despite recognizing that these models were clearly a simplification of reality. We then used extensive simulation testing to evaluate how well our approach performed when data were generated under a variety of more complicated (and realistic) scenarios. This general approach of using simpler models, with extensive simulation testing, largely contrasts with the current trend in ecology to fit rather complex models, often with hierarchical specifications involving random effects, or state-space models that attempt to separately model observation and process components [12], [33], [34]. The latter require sophisticated numerical integration routines, approximate likelihood techniques, or Bayesian approaches that utilize Markov Chain Monte Carlo (MCMC) methods for parameter estimation. In such cases, long computation time for model fitting typically limits the amount of simulation testing that can be accomplished. Simulation testing is further complicated by parameter identifiability issues often associated with age-at-harvest models, which may cause Frequentist procedures to fail to converge. Similarly, Bayesian applications require careful inspection of MCMC samplers for proper convergence and additional time should be devoted to exploring sensitivity of posterior estimates to prior distributional assumptions.

We believe our general approach was useful for initial model development and testing, and offers a foundation on which future modeling efforts can be built. A logical next step would be to consider models that incorporate random effects to describe temporal (process) variability in harvest and survival rates. Allowing the yearly vulnerability parameters in the *H*(*a*, *s*, *yr*) models to be modeled as random effects would shrink them towards their overall mean, and would likely lead to much more precise (and reliable) estimates in later years when there is less information available for estimating harvest vulnerabilities. Random effects could also be added to the *H*(*a*, *s*, *f*, *e*) models to allow for additional variability in harvest vulnerabilities not attributable to food availability or hunting effort, adding flexibility and biological realism to these models. Our application benefited by the fact that harvest was the predominant source of mortality (e.g., ∼80% of 330 radio-collared bears that died during 1981–2008 were killed by hunters; MNDNR unpublished data). However, our models were fairly aggressive with respect to the number of estimated parameters relative to the amount of available information in the data, with the ratio of “cells” (representing unique sex×age×year combinations) to parameters roughly equal to 1∶7 for the *H*(*a*, *s*, *yr*) models and 1∶9 for the *H*(*a*, *s*, *f*, *e*) models. One option to reduce the number of parameters would be to constrain the initial age distributions (e.g., by estimating starting population sizes for males and females and a limited number of parameters that describe the proportion of individuals falling into each age class; see e.g.,[35]). Another option would be to estimate a small number of fecundity parameters rather than directly estimate the number of cubs in each year, but this change might also require additional state variables (mothers with and without cubs) since mothers with cubs generally forgo reproduction.

Lastly, we would argue that additional efforts are needed to explore the reliability of various methods for characterizing uncertainty in population trends estimated from fitted models. A few different approaches have been taken in the applied literature. Gove et al. [2] used asymptotic quasi-likelihood based intervals, in which standard errors derived from the inverse of the Hessian matrix were inflated using χ^2^/df, where df = (number of ages×number of years−number of estimated parameters). We used a χ^2^ objective function for model fitting, and similar asymptotic arguments may be used to construct confidence intervals in this case [36]. Others have used bootstrapping and Monte Carlo approaches [8], which we illustrate in our application. Finally, Bayesian applications naturally characterize uncertainty using posterior distributions for model parameters [6]. It is unclear how well any of these methods will perform in cases where the estimation model is a gross simplification of reality (as it often will be), or when certain parameters are assumed to be known without error. Our bootstrap intervals were extremely narrow for many of our model-based predictions (e.g., Figure 6), and they do not account for arguably the greatest source of uncertainty, namely that resulting from approximating the underlying true population dynamics with a simplified process model. At best, these intervals should be thought of as “variability bands,” describing how the model might perform if the data collection process could be repeated rather than an interval that is likely to contain true parameters 95% of the time. Similarly, it is unclear how well posterior credibility intervals will perform when distributional assumptions are not met, when prior distributions are unavoidably informative, or when parameters are only weakly indentified. Unfortunately, the performance of these methods for characterizing uncertainty is likely to depend on both model and data, and testing these methods will also require extensive computations.

We end with a quote, “You have a big approximation and a small approximation. The big approximation is your approximation to the problem you want to solve. The small approximation is involved in getting the solution to the approximate problem.” [Doug Bates recalls George Box saying this (D. Bates, personal communication).] In the context of our applied problem, we viewed the task of building a model to reflect the underlying population and harvest dynamics as the big approximation and the approach to model fitting as the small approximation. By choosing a modeling approach simple enough for extensive simulation testing tailored to our available data, we were able to focus on the implications of our big approximation. Including random effects or making use of state-space models would allow us to fit more realistic population models (i.e., giving a better big approximation). Unfortunately the difficulty in fitting such models often limits our ability to evaluate the effect of the big approximation in real-world dynamics. We believe this tradeoff is important to consider in many modeling applications.

## Supporting Information

Box S1 List of estimated parameters for the *H*(*a*, *s*, *f*, *e*) and *H*(*a*, *s*, *yr*) sub-models.
(0.03 MB PDF)

Table S1 Summary of simulation results for each of the six estimators in all eight simulation scenarios.
(0.05 MB PDF)

Figure S1 Time series of true and estimated abundances, scaled by the inverse of the overall mean across the time series, for each of the six estimators in all eight simulation scenarios.
(0.08 MB PDF)

Appendix S1 A summary of natural food availability, hunter effort, and telemetry data considered in the integrated population analysis of black bears in Minnesota.
(0.19 MB PDF)

Appendix S2 A description of the simulation study (methods) used to test the robustness of the integrated population modeling approach.
(0.07 MB PDF)

Appendix S3 Additional table and figures summarizing the fits of integrated population models to Minnesota black bear data.
(0.10 MB PDF)

Appendix S4 A .zip file containing an AD Model Builder executable, a data input file (bbM2b.dat), an R file (modrun.R), and other associated files needed to fit the H(a, s, f, e) models to the Minnesota black bear data.
(1.19 MB ZIP)
