# Supplementary material for: Integrated Population Modeling of Black Bears in Minnesota: Implications for Monitoring and Management
Source: PLoS One. 2010 Aug 12;5(8):e12114. doi: 10.1371/journal.pone.0012114 (PMC2920827; doi:10.1371/journal.pone.0012114)
Supplement: Box S1 — List of estimated parameters for the H(a, s, f, e) and H(a, s, yr) sub-models. (0.03 MB PDF) [file pone.0012114.s001.pdf]

Box S1

List of estimated parameters for the *H*(*a, s, f, e*) and *H*(*a, s*, *yr*) sub-models

H(*a,s,f,e*) sub-models (total parameters = 63):

- Starting population sizes in 1980 for both sexes and all age classes (cubs, ages 1-9, age 10+) = 22 estimated parameters
- Number of cubs, $N_{t,0}$, in 1981-2007 = 27 parameters
  note: $N_{1980,\,0}$ is included in the starting population size
  $N_{2008,\,0,}$ is not estimated (these bears are never harvested so there is no information in the data to estimate this parameter)
- Survival of yearling females (relative to male yearlings) = 1 parameter
- Survival for male and female bears age 2 and older = 2 parameters
- Harvest parameters used to model temporal variability:
  - Vulnerabilities in 1980-1983 (where food index and hunting effort data were lacking) = 4 parameters
  - Regression parameters for years 1985-2008 reflecting an overall intercept and effects of food availability and hunting effort = 3 parameters
- Harvest regression parameters to model effect of sex and age (3 df) = 4 parameters

H(*a, s, yr*) sub-models (total parameters = 85):

- Starting population sizes in 1980 for both sexes and all age classes (cubs, ages 1-9, age 10+) = 22 estimated parameters
- Number of cubs, $N_{t,0}$, in 1981-2007 = 27 parameters
- Survival of yearling females (relative to male yearlings) = 1 parameter
- Survival for male and female bears age 2 and older = 2 parameters
- Harvest rate parameters used to model temporal variability:
  - Vulnerabilities in 1980-2008 =29 parameters
- Harvest regression parameters to model effect of sex and age (3 df) = 4 parameters
