# Supplementary material for: Integrated Population Modeling of Black Bears in Minnesota: Implications for Monitoring and Management
Source: PLoS One. 2010 Aug 12;5(8):e12114. doi: 10.1371/journal.pone.0012114 (PMC2920827; doi:10.1371/journal.pone.0012114)
Supplement: Table S1 — Summary of simulation results for each of the six estimators in all eight simulation scenarios. (0.05 MB PDF) [file pone.0012114.s002.pdf]

Table S1. Summary of simulation results for each simulation scenario and estimator (defined by the harvest model parameterization and penalty weights for the mark-recapture component of the objective function). Bold type indicates, for each scenario, estimation models that yielded unbiased estimates of population size (3rd column) or had the smallest MSE values (4th column).

Scenario[1]	Estimator[2]	Mean $\hat{N}$ (thousands)[3]	MSE$\left(\hat{\lambda}_t\right)$[4]	$\hat{\bar{S}}_T(Ad_M, Yr_F, Ad_F)$[5]	No. npd[6]
Baseline	*H*(*a*, *s*, *f*, *e*; *w*=0)	14.09	$5.0E^{-5}$	(0.92, 0.92, 0.97)	0
True $\bar{N}$[7] = 13.96	*H*(*a*, *s*, *f*, *e*; *w*=1)	14.01	**$3.6E^{-5}$**	(0.92, 0.92, 0.97)	0
	H(*a*, *s*, *f*, *e*; *w*=200)	**13.98**	$1.3E^{-4}$	(0.92, 0.92, 0.97)	1
$\bar{S}_T(Ad_M, Yr_F, Ad_F)$[8]	*H*(*a*, *s*, *yr*; *w*=0)	15.33	$2.2E^{-4}$	(0.91, 0.93, 0.96)	0
(0.92, 0.92, 0.97)	*H*(*a*, *s*, *yr*; *w*=1)	14.04	$6.4E^{-5}$	(0.92, 0.92, 0.97)	0
	H(*a*, *s*, *yr*; *w*=200)	14.02	$5.4E^{-4}$	(0.92, 0.92, 0.97)	0
	Downing males	NA	$9.6E^{-3}$	NA	NA
	Downing females	NA	$9.2E^{-3}$	NA	NA
	Downing both	NA	$9.2E^{-3}$	NA	NA
Stochastic Rates	*H*(*a*, *s*, *f*, *e*; *w*=0)	16.71	0.0021	(0.91, 0.94, 0.96)	0
True $\bar{N}$ = 11.71	*H*(*a*, *s*, *f*, *e*; *w*=1)	12.31	**0.0016**	(0.93, 0.93, 0.98)	2
	H(*a*, *s*, *f*, *e*; *w*=200)	**11.67**	0.0017	(0.94, 0.92, 0.98)	6
$\bar{S}_T(Ad_M, Yr_F, Ad_F)$	*H*(*a*, *s*, *yr*; *w*=0)	29.12	0.0043	(0.88, 0.95, 0.94)	2
(0.92, 0.92, 0.97)	*H*(*a*, *s*, *yr*; *w*=1)	12.05	0.0022	(0.94, 0.93, 0.98)	4
	H(*a*, *s*, *yr*; *w*=200)	**11.71**	0.0021	(0.94, 0.93, 0.98)	8
	Downing males	NA	0.0140	NA	NA
	Downing females	NA	0.0130	NA	NA
	Downing both	NA	0.0100	NA	NA
Trend in Harvest	*H*(*a*, *s*, *f*, *e*; *w*=0)	15.55	$7.1E^{-4}$	(0.86, 0.94, 0.93)	0
True $\bar{N}$ = 11.96	*H*(*a*, *s*, *f*, *e*; *w*=1)	12.01	$4.1E^{-4}$	(0.90, 0.90, 0.96)	0
	H(*a*, *s*, *f*, *e*; *w*=200)	11.61	$5.6E^{-4}$	(0.91, 0.90, 0.96)	3
$\bar{S}_T(Ad_M, Yr_F, Ad_F)$	*H*(*a*, *s*, *yr*; *w*=0)	13.56	$3.0E^{-4}$	(0.91, 0.95, 0.96)	17
(0.92, 0.92, 0.97)	*H*(*a*, *s*, *yr*; *w*=1)	**11.97**	**$6.8E^{-5}$**	(0.92, 0.93, 0.97)	0
	H(*a*, *s*, *yr*; *w*=200)	12.02	$5.6E^{-4}$	(0.92, 0.91, 0.97)	3
	Downing males	NA	$1.0E^{-2}$	NA	NA
	Downing females	NA	$9.9E^{-3}$	NA	NA
	Downing both	NA	$9.9E^{-3}$	NA	NA
Incorrect Survival	*H*(*a*, *s*, *f*, *e*; *w*=0)	10.87	$9.3E^{-5}$	(0.94, 0.88, 0.96)	0
True $\bar{N}$ = 10.57	*H*(*a*, *s*, *f*, *e*; *w*=1)	10.67	**$6.9E^{-5}$**	(0.94, 0.88, 0.96)	0
	H(*a*, *s*, *f*, *e*; *w*=200)	**10.58**	$1.7E^{-4}$	(0.94, 0.88, 0.97)	1
$\bar{S}_T(Ad_M, Yr_F, Ad_F)$	*H*(*a*, *s*, *yr*; *w*=0)	12.20	$3.1E^{-4}$	(0.92, 0.89, 0.95)	0
(0.92, 0.84, 0.97)	*H*(*a*, *s*, *yr*; *w*=1)	10.72	$1.0E^{-4}$	(0.94, 0.88, 0.96)	0
	H(*a*, *s*, *yr*; *w*=200)	10.62	$5.4E^{-4}$	(0.94, 0.88, 0.97)	0

	Downing males	NA	8.9E^{-3}	NA	NA
	Downing females	NA	8.5E^{-3}	NA	NA
	Downing both	NA	8.5E^{-3}	NA	NA
Reporting Error	*H*(*a*, *s*, *f*, *e; w*=0)	14.65	1.7E^{-4}	(0.93, 0.98, 0.97)	9
True $\overline{N}$ = 13.95	*H*(*a*, *s*, *f*, *e; w*=1)	14.14	**1.4 E^{-4}**	(0.94, 0.98, 0.97)	19
	H(*a*, *s*, *f*, *e; w*=200)	13.82	2.1 E^{-4}	(0.95, 0.97, 0.98)	5
$\overline{S}_T(Ad_M, Yr_F, Ad_F)$	*H*(*a*, *s*, *yr; w*=0)	13.05	3.2 E^{-4}	(0.96, 0.96, 0.99)	2
(0.92, 0.92, 0.97)	*H*(*a*, *s*, *yr; w*=1)	13.47	3.0 E^{-4}	(0.95, 0.96, 0.98)	2
	H(*a*, *s*, *yr; w*=200)	13.69	8.9 E^{-4}	(0.94, 0.96, 0.98)	3
	Downing males	NA	9.7 E^{-3}	NA	NA
	Downing females	NA	9.2 E^{-3}	NA	NA
	Downing both	NA	9.2 E^{-3}	NA	NA
Food x Sex Interaction	*H*(*a*, *s*, *f*, *e; w*=0)	21.38	5.4E^{-5}	(0.92, 0.96, 0.97)	39
True $\overline{N}$ = 22.31	*H*(*a*, *s*, *f*, *e; w*=1)	22.22	**4.4E^{-5}**	(0.92, 0.92, 0.97)	9
	H(*a*, *s*, *f*, *e; w*=200)	**22.35**	1.2E^{-4}	(0.92, 0.92, 0.97)	19
$\overline{S}_T(Ad_M, Yr_F, Ad_F)$	*H*(*a*, *s*, *yr; w*=0)	20.04	1.3E^{-4}	(0.92, 0.94, 1.00)	30
(0.92, 0.92, 0.97)	*H*(*a*, *s*, *yr; w*=1)	22.05	1.1E^{-4}	(0.92, 0.89, 0.99)	8
	H(*a*, *s*, *yr; w*=200)	22.57	2.5E^{-4}	(0.92, 0.94, 0.97)	16
	Downing males	NA	5.9E^{-3}	NA	NA
	Downing females	NA	4.6E^{-3}	NA	NA
	Downing both	NA	4.1E^{-3}	NA	NA
Increasing S(*t*)	*H*(*a*, *s*, *f*, *e; w*=0)	13.93	5.8E^{-5}	(0.91, 0.90, 0.97)	0
True $\overline{N}$ = 13.37	*H*(*a*, *s*, *f*, *e; w*=1)	13.47	**4.8E^{-5}**	(0.92, 0.90, 0.97)	0
	H(*a*, *s*, *f*, *e; w*=200)	13.26	1.6E^{-4}	(0.92, 0.89, 0.98)	2
$\overline{S}_T(Ad_M, Yr_F, Ad_F)$	*H*(*a*, *s*, *yr; w*=0)	13.98	1.5E^{-4}	(0.92, 0.90, 0.97)	1
(0.92, 0.92, 0.97)	*H*(*a*, *s*, *yr; w*=1)	**13.37**	9.9E^{-5}	(0.92, 0.90, 0.98)	0
	H(*a*, *s*, *yr; w*=200)	13.29	5.6E^{-4}	(0.92, 0.90, 0.98)	3
	Downing males	NA	1.0E^{-2}	NA	NA
	Downing females	NA	9.3E^{-3}	NA	NA
	Downing both	NA	9.4E^{-3}	NA	NA
Kitchen Sink	*H*(*a*, *s*, *f*, *e; w*=0)	16.54	1.1E^{-3}	(0.91, 0.90, 0.97)	18
True $\overline{N}$ = 12.33	*H*(*a*, *s*, *f*, *e; w*=1)	11.79	9.6E^{-4}	(0.92, 0.91, 0.97)	3
	H(*a*, *s*, *f*, *e; w*=200)	12.13	9.0E^{-4}	(0.93, 0.91, 0.97)	2
$\overline{S}_T(Ad_M, Yr_F, Ad_F)$	*H*(*a*, *s*, *yr; w*=0)	11.05	7.6E^{-4}	(0.92, 0.90, 0.99)	23
(0.94, 0.84, 0.97)	*H*(*a*, *s*, *yr; w*=1)	11.75	**6.9E^{-4}**	(0.93, 0.89, 0.98)	0
	H(*a*, *s*, *yr; w*=200)	12.44	8.2E^{-4}	(0.93, 0.91, 0.97)	6
	Downing males	NA	1.1E^{-2}	NA	NA
	Downing females	NA	8.8E^{-3}	NA	NA

Downing both	NA	$6.7E^{-3}$	NA	NA

[1] The simulation scenarios, described in more detail earlier in this Appendix, are listed in italics.

[2] *H*(*a, s, f, e*) estimators model temporal variability in harvest rates as a function of food availability and hunting effort indices, whereas the *H*(*a, s, f, yr*) estimators use an unstructured model for harvest rates. In both cases, *w* refers to the weight assigned to the mark-recapture component of the objective function used to fit the model.

[3] Mean estimate of abundance (averaged across years and simulation runs) for each estimator, with values in bold representing those cases in which the mean estimate was within Monte Carlo error of the true mean abundance.

[4] Mean squared error (MSE), multiplied by 1000, between true and estimated yearly transitions ($\lambda_t = N_{t+1} / N_t$) across years and simulation runs = $\sum_{j=1}^{1000}\sum_{i=1}^{28}\left(\lambda_{i,j} - \hat{\lambda}_{i,j}\right)^2 / 28$.

[5] Mean survival (from non-hunting mortality sources) for adult males (Ad_M), yearling females (Yr_F), and adult females (Ad_F).

[6] Number of simulations (out of 1000) in which the Hessian matrix was non-positive definite (indicating that a minimum was not found).

[7] True $\overline{N}$ = mean (true) abundance (in thousands) over the 29 year time series (and across the 1000 simulation runs).

[8] $\overline{S}_T(Ad_M, Yr_F, Ad_M)$ = survival rates by age class (averaged across years and simulation runs).
