# Supplementary material for: Integrated Population Modeling of Black Bears in Minnesota: Implications for Monitoring and Management
Source: PLoS One. 2010 Aug 12;5(8):e12114. doi: 10.1371/journal.pone.0012114 (PMC2920827; doi:10.1371/journal.pone.0012114)
Supplement: Figure S1 — Time series of true and estimated abundances, scaled by the inverse of the overall mean across the time series, for each of the six estimators in all eight simulation scenarios. (0.08 MB PDF) [file pone.0012114.s003.pdf]

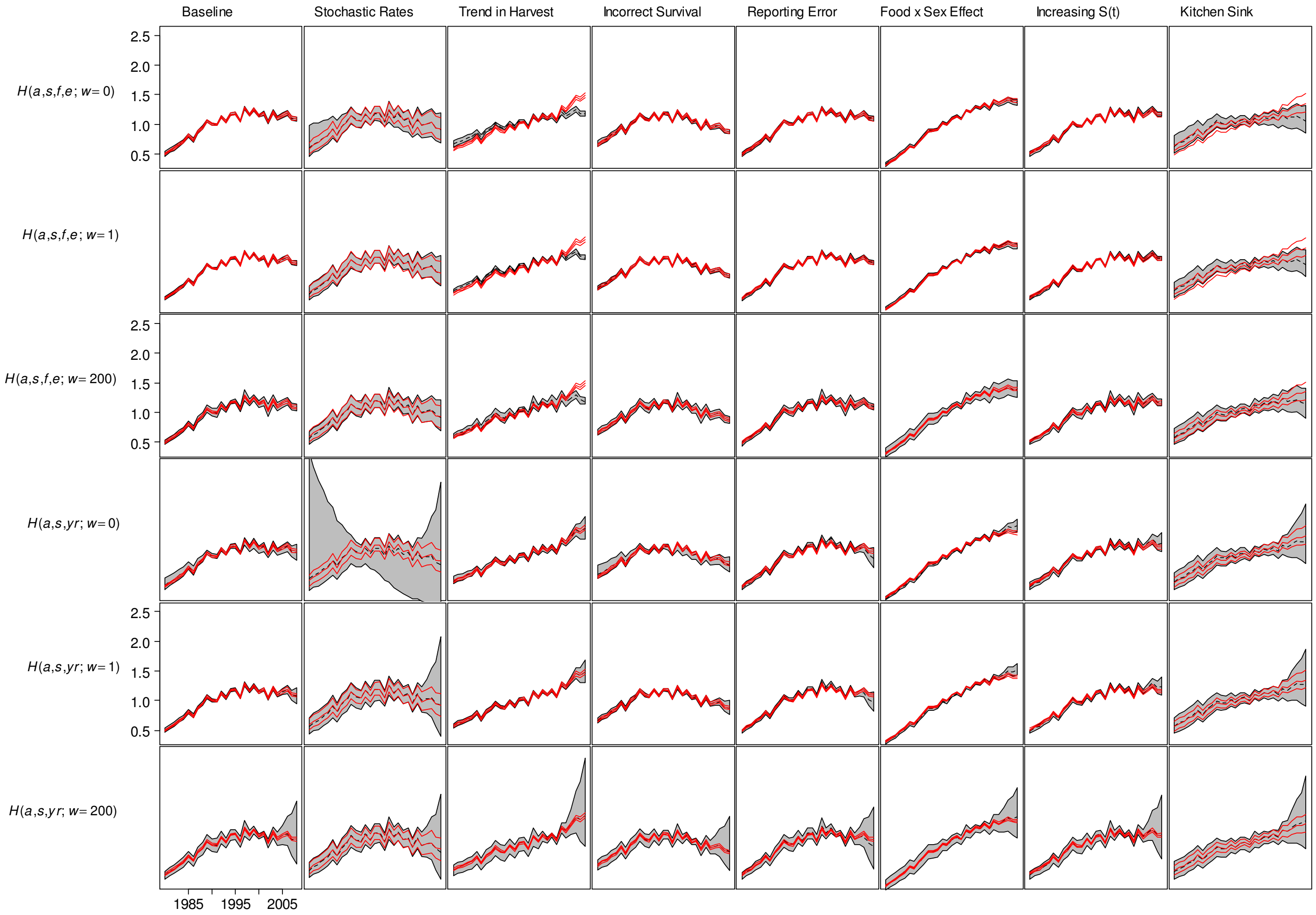

Figure S1. Performance of age-at-harvest abundance estimators with simulated data. In each panel, the y-axis depicts abundance, scaled by the inverse of the overall mean across the time series. Red lines correspond to (2.5th, 50th, and 97.5th percentiles) of the true population dynamics (across stochastic simulations). Gray polygon encompasses 95% of the estimated values. Rows correspond to different estimators and columns correspond to different simulation scenarios.
