# Supplementary material for: Integrated Population Modeling of Black Bears in Minnesota: Implications for Monitoring and Management
Source: PLoS One. 2010 Aug 12;5(8):e12114. doi: 10.1371/journal.pone.0012114 (PMC2920827; doi:10.1371/journal.pone.0012114)
Supplement: Appendix S1 — A summary of natural food availability, hunter effort, and telemetry data considered in the integrated population analysis of black bears in Minnesota. (0.19 MB PDF) [file pone.0012114.s004.pdf]

Appendix S1: Auxiliary Data

Independent data on mortality of Minnesota bears were obtained from a long-term telemetry-based study that we have conducted since 1981 at a site within the Chippewa National Forest (CNF), near the center of Minnesota's primary bear range (Figure S1.1). This site is representative of a broad area of the range in terms of habitat and hunting pressure. We also initiated other telemetry studies along the southern (1991), northern (1997) and western (2007) edges of the range in order to collect data that encompass the full variation of habitat features, foods, and hunting pressures within Minnesota's black bear population (Garshelis and Noyce 2008). All of these studies involved radio-collaring bears ($n > 500$ total among all study sites), with primary objectives centering on variation in reproduction and mortality (by bear age, geographic area, habitat, food conditions, and year). However, here we focus on data from the CNF because it includes the longest time series, the largest sample of bears, and represents a large portion of Minnesota's bear range.

Use of auxiliary data in the MN Black Bear application and in the simulation study
We used estimates of cub survival rates as (fixed) inputs to our estimation models (both in the simulation study and in the MN black bear applications). We used statewide indices of food availability and hunting effort (Table S1.1) to model temporal variability in harvest rates in the MN black bear application. We used harvest mortality data for radio-collared bears and food availability and hunting effort indices measured at a more local scale (i.e., representative of the CNF study site; Table S1.2) to derive parameter inputs for the operating models used in the simulation study (Appendix S2). We also compared harvest rates (as a function of age, sex, food availability, and hunting effort) estimated from radio-collared bears to those obtained from applying integrated population models to our MN age-at-harvest data (as an independent test of model reliability). Lastly, we used estimates of adult survival rates to derive inputs for the operating models used in the simulation study.

Mortality of bears older than cubs
Bears were captured in traps, radio-collared, and most were monitored until they died. All radio collars contained a switch that changed the pulse mode of the radio signal if the collar did not move for 4 hours, an indication that the bear had died. To detect mortalities, radio signals of bears were monitored approximately weekly from an airplane during the first 10 years of the study, and less often after that.

Hunting was the primary source of mortality for radio-collared bears: 229 of 279 (82%) collared bears in the CNF with known causes of mortality were killed by hunters (see Table S1.2 and Figure S1.2 for summaries of the harvest data from this study site for years 1982-2004). Hunting collared bears was legal, and hunters were directed to treat them like any other bear; radio collars were black in color so most hunters did not see them until the bear was killed. Hunters generally complied in reporting the killing of collared bears and returning their collars, and were given a small reward. Hunters were required to register all bears with the Minnesota Department of Natural Resources (MNDNR) and report the date of kill, so this was known exactly for most harvested radio-collared bears.

Cub mortality
Females gave birth in January, generally every other year beginning at 4 or 5 years old (Garshelis et al. 1998). Radio-collared females were tracked to their dens in March to assess reproduction. They were anesthetized and removed from the den. Cubs were not anesthetized but were sexed and ear-tagged. No known mortality occurred from handling cubs or replacing them back with their mother. The same females were checked in dens the following year. Cubs remained with their mother for 17 months, so their absence in their mother's den the following winter indicated that they had died (none were ever subsequently recovered). Dates of cub deaths were unknown, so survival rates were estimated simply as the proportion observed denning with their mother as yearlings (1-year-olds). At the CNF study site, 180 male and 171 female cubs were observed in natal dens from March 1982 to March 2008, of which 136 males and 151 females survived their first year, yielding estimated survival rates of 0.76 and 0.88 for male and female cubs, respectively. Mothers and yearling bears were anesthetized in dens, and yearlings were radio-collared for subsequent monitoring.

Food availability
Natural food availability (mainly fruits and nuts) affects harvest rates of bears because most Minnesota hunters attempt to attract bears with bait: bears are less attracted to hunters' baits when natural foods are plentiful (Noyce and Garshelis 1997, Garshelis and Noyce 2008). Field personnel from across the state (n = 40–50 each year) subjectively scored the productivity of fruits eaten by bears on a scale of 0–4 (2 = average, 4 = very abundant). Observations of bears indicated that three fall fruits (dogwood berries [*Cornus* spp.], hazelnuts [*Corylus* spp.], and acorns [*Quercus* spp.]) comprised most of their diet during the hunting season, and productivity of these fruits most affected hunter harvest (Noyce and Garshelis 1997). Consequently, we included only the yearly scores representing the sum of these three fruits when deriving food availability indices statewide (Table S1.1) or in the general area used by collared bears in the CNF study site (Table S1.2).

Hunting effort
Hunting pressure on Minnesota bears was regulated by the MNDNR through yearly revisions to quotas on the number of available hunting licenses that were aimed at effecting changes in population size. Hunters were required to purchase a license to hunt bears within a specified geographic management zone. Licenses had to be purchased well in advance of the hunting season, so not all license-holders were actually able to hunt. Periodic surveys of licensed hunters by the MNDNR provided estimates of the proportion that actually hunted each year. We multiplied licenses sold by estimates of the proportion hunting (86.8–93.9%) to derive estimates of the number of people hunting each year, both statewide (Table S1.1) and within the management zones occupied by bears in the CNF study site (Table S1.2). We had incomplete information on the number of days hunters spent hunting each year, so could not incorporate this into the measure of hunting effort.

The bear hunting season began on September 1 and lasted until mid-October (6–7 weeks), with most of the hunting pressure early in the season (on average, ~70% of the total harvest occurred during the first week of the season; Garshelis and Noyce 2010). Thus, season length, which varied by a few days each year, did not affect harvest pressure.

Estimation of harvest mortality rates

We treated the harvest mortality data for collared bears as though they were interval censored (Kalbfleisch and Prentice 2002), with intervals defined by each hunting season. This simplification allowed us to include bears that died during the hunting season but whose exact date of death was unknown. We assumed the data follow a continuous time proportional hazards model:

$$\lambda_i(t \mid x_i(t)) = \lambda_o(t)\exp\{x_i(t)\beta\}, \qquad \text{(A1)}$$

where $\lambda_i(t \mid x_i(t))$ is the hazard (or instantaneous risk of death) at time t for individual i, $x_i(t)$ is a vector of covariates for individual i and β is a vector of regression parameters. The term $\lambda_o(t)$ gives the baseline hazard at time t (equivalent to the hazard for an individual with all covariates = 0). We considered two individual-level covariates, age and sex. We updated age each year, and modeled its effect using natural cubic regression splines with 3 degrees of freedom [interior knots were set to ages (2, 7) and outer knots were set to (1,10)]. Under this assumed model, the probability of subject i dying in the jth interval, given that it was alive at the start of this interval, $\pi_{i,j}$, is given by (Kalbfleisch and Prentice 1973, 2002):

$$\pi_{i,j} = 1 - \exp(-\exp(\alpha_j + \vec{x}_{i,j}^T\vec{\beta})), \qquad \text{(A2)}$$

where the α_j's allow the baseline risk to vary by interval, $x_{i,j}$ is a (4 x 1) vector containing the individual-level predictors for subject i during interval j (i.e., the spline basis function for age and an indicator variable for sex) and β is a (4 x 1) vector of regression parameters. We further assumed, based on regression models that predicted the total statewide harvest from an index of fall food abundance and hunter numbers (25 years of data, $R^2 = 0.86$; Garshelis and Noyce 2010), that the interval specific baseline hazards in year j, α_j, could be modeled as a linear function of this food availability index (f_j) and hunting effort (e_j):

$$\alpha_j = \alpha_o + \gamma_1 \cdot f_j + \gamma_2 \cdot e_j \qquad \text{(A3)}$$

We fit this model using the glm function in R (R Development Core Team 2009), specifying a complementary log-log link. Results of the fitted model are given below (see also Figure 2 of the main text):

```
                               Estimate Std. Error z value Pr(>|z|)
(Intercept)                    -0.50567    0.43443  -1.164  0.24443
sex                             0.37005    0.14312   2.586  0.00972 **
hunters                         0.05280    0.02777   1.901  0.05727 .
ns(ages, 3, knots = c(2, 7))1 -0.53678    0.53508  -1.003  0.31578
ns(ages, 3, knots = c(2, 7))2 -1.25771    0.65776  -1.912  0.05586 .
ns(ages, 3, knots = c(2, 7))3 -1.37073    1.20442  -1.138  0.25508
foodf                          -0.16816    0.06302  -2.668  0.00762 **
---
Signif. codes:  0 '***' 0.001 '**' 0.01 '*' 0.05 '.' 0.1 ' ' 1

(Dispersion parameter for binomial family taken to be 1)
```

```
    Null deviance: 1063.6  on 1019  degrees of freedom
Residual deviance: 1036.7  on 1013  degrees of freedom
AIC: 1050.7
```

For the simulation study, we also fit a model in which the effect of food interacted with sex. A summary of the fitted coefficients for this extended model is given below (see also Figure S2.4 in Appendix S2):

```
Coefficients:
                              Estimate Std. Error z value Pr(>|z|)
(Intercept)                    0.11469    0.58471   0.196   0.8445
sex                           -0.87188    0.80124  -1.088   0.2765
hunters                        0.05180    0.02783   1.861   0.0627 .
ns(ages, 3, knots = c(2, 7))1 -0.55526    0.53468  -1.038   0.2990
ns(ages, 3, knots = c(2, 7))2 -1.26134    0.65469  -1.927   0.0540 .
ns(ages, 3, knots = c(2, 7))3 -1.33445    1.19822  -1.114   0.2654
foodf                         -0.26447    0.08849  -2.989   0.0028 **
sex:foodf                      0.19722    0.12500   1.578   0.1146
---
Signif. codes:  0 '***' 0.001 '**' 0.01 '*' 0.05 '.' 0.1 ' ' 1

(Dispersion parameter for binomial family taken to be 1)

    Null deviance: 1063.6  on 1019  degrees of freedom
Residual deviance: 1034.1  on 1012  degrees of freedom
AIC: 1050.1
```

Estimation of adult survival rates

Using the telemetry data from the CNF study site, we modeled survival of male and female bears (age ≥ 2) separately, assuming the risk of mortality varied smoothly throughout the year and that this seasonal trend was consistent from year to year (referred to as a recurrent model in Fieberg and DelGiudice 2009). We divided the follow-up time for each individual into 3000 intervals of constant duration using the "split-Lexis" function of the Epi R package (Carstensen et al. 2008). We then modeled survival rates as a non-linear function of Julian date on the log scale, using regression splines with 3 degrees of freedom to model the seasonal time trend, with the duration of the follow-up interval included as an offset (Carstensen et. al. 2006a,b). Following the methods outlined in Carstensen (2006a,b), we estimated survival rates from 26 April to 1 September (the start of the hunting season) by first estimating survival for each of the shorter time intervals and then taking their product, using the "ci.cum" function in the Epi package (Carstensen et al. 2008).

Literature Cited

Carstensen B, Plummer M, Laara E, Hills M (2008). Epi: A package for statistical analysis in epidemiology. R package version 1.0.8. Available http://www.pubhealth.ku.dk/~bxc/Epi/ via the internet. Accessed 2009 December 20.

Carstensen B (2006a) Demography and epidemiology: Age-Period-Cohort models in the computer age. Technical Report 06.1, Department of Biostatistics, University of Copenhagen, Copenhagen, Denmark. Available: http://biostat.ku.dk/reports/2006/ via the internet. Accessed 2009 September 8.

Carstensen B (2006b) Demography and epidemiology: practical use of the Lexis diagram in the computer age. Or: Who needs the Cox-model anyway? Technical Report 06.2, Department of Biostatistics, University of Copenhagen, Copenhagen, Denmark. Available: http://biostat.ku.dk/reports/2006/ via the internet. Accessed 2009 September 8.

Fieberg J, DelGiudice GD (2009) What time is it? Choice of time origin and scale in extended proportional hazards models. Ecology 90:1687-1697.

Garshelis, DL, Noyce KV (2008) Seeing the world through the nose of a bear — Diversity of foods fosters behavioral and demographic stability. In Fulbright T, Hewitt D, editors, Frontiers in Wildlife Science: Linking Ecological Theory and Management Applications. Boca Raton: CRC Press. pp. 139–163.

Garshelis DL, Noyce KV (2010) Status of Minnesota black bears, 2009. Final report to bear committee. Minnesota Department of Natural Resources.

Garshelis DL, Noyce KV, Coy PL (1998) Calculating average age of first reproduction free of the biases prevalent in bear studies. Ursus 10:437-447.

Kalbfleisch JD, Prentice RL (1973) Marginal likelihoods based on Cox's regression and life model. Biometrika 60:267-278.

Kalbfleisch JD, Prentice RL (2002) The statistical analysis of failure time data, 2nd Edition. Hoboken: John Wiley & Sons. 462 p.

Noyce KV, Garshelis DL (1997) Influence of food abundance on black bear harvests in Minnesota. J Wildl Manage 61:1067-1074.

R Development Core Team (2009) R: A Language and Environment for Statistical Computing. R Foundation for Statistical Computing, Vienna, Austraia. Available: http://www.R-project.org via the intranet. Accessed 2009 August 31.

Table S1.1. Statewide food and hunting effort indices used to model temporal variability in harvest rates in the integrated population models applied to Minnesota black bear age-at-harvest data.

Year	Estimated number of hunters	Fall food index[1]
1984	3,100	6.5
1985	3,700	4.4
1986	3,900	6.2
1987	5,600	7.7
1988	5,100	6.7
1989	5,500	5.8
1990	6,600	5.2
1991	7,200	6.7
1992	7,900	5.1
1993	8,600	6.5
1994	9,100	7.2
1995	11,600	4.9
1996	11,500	8.6
1997	10,300	6.2
1998	14,500	6.7
1999	15,900	6.2
2000	16,800	7.0
2001	15,500	5.2
2002	13,700	8.1
2003	13,500	6.1
2004	12,800	5.9
2005	12,400	6.2
2006	12,400	6.3
2007	11,200	6.2
2008	9,800	7.1

[1] Sum of scores of three key foods, ranked on a scale of 0–4 (average summed production = 2 *x* 3 fruits = 6; maximum production = 4 *x* 3 = 12). Note: food surveys in 1981–83 were based on data collected somewhat differently than the other years, so they were not included in the integrated population models.

Table S1.2. Raw data on hunter harvests of radio-collared bears from the Chippewa National Forest (CNF) study site, with associated key variables affecting harvest rates.

Year	Estimated number of hunters	Fall food index[1]	Number bears survived	Number bears killed by hunters
1982	1,132	6.00	32	3
1983	2,180	8.00	27	5
1984	2,241	6.65	41	12
1985	2,547	4.38	44	8
1986	2,645	6.23	49	16
1987	2,429	7.71	58	13
1988	2,496	6.73	59	19
1989	2,777	5.75	69	20
1990	3,620	5.45	50	24
1991	4,113	6.95	55	6
1992	4,590	5.37	48	16
1993	5,208	6.82	34	14
1994	5,797	7.55	38	10
1995	7,207	4.84	30	15
1996	7,360	8.80	45	5
1997	6,992	6.38	13	4
1998	10,088	6.80	11	1
1999	11,706	6.09	7	2
2000	12,011	7.70	13	3
2001	10,148	5.47	15	3
2002	9,051	8.14	20	3
2003	8,918	6.20	17	8
2004	8,213	6.02	12	5
2005[2]	7,819	6.48	13	5

[1] Sum of scores of three key foods, ranked on a scale of 0–4 (average summed production = 2 *x* 3 fruits = 6; maximum production = 4 *x* 3 = 12).

[2] Data set was truncated after 2005 due to a diminishing sample of radio-collared bears. Also, study objectives changed and an effort was made to dissuade hunters from shooting the remaining collared bears.

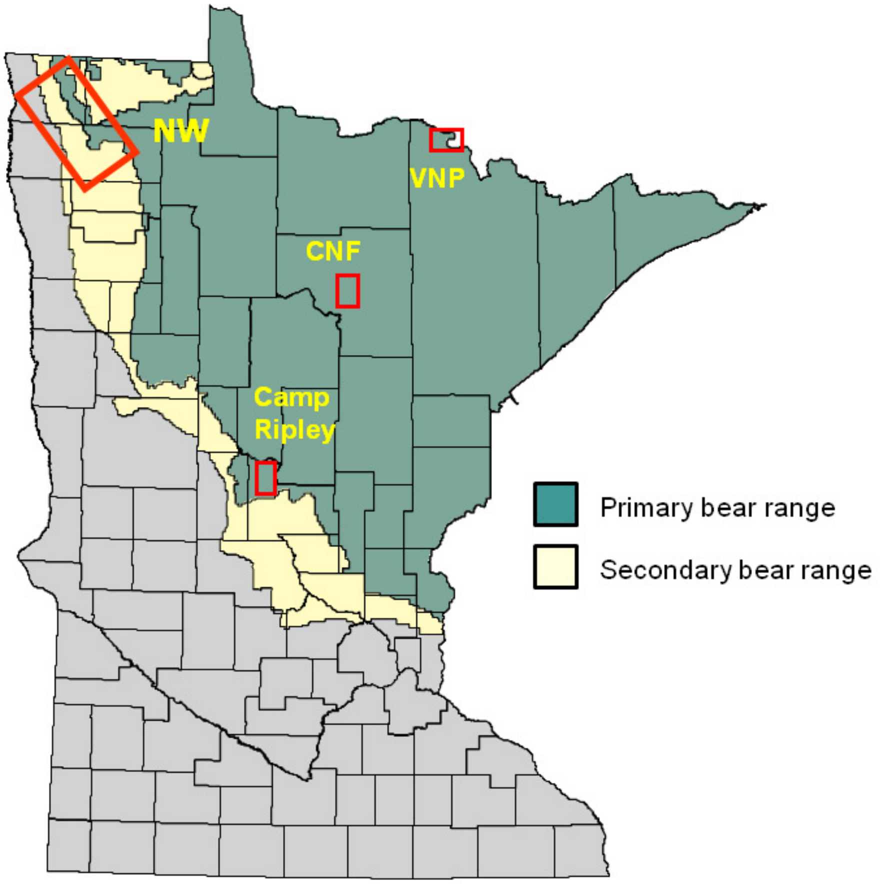

Figure S1.1. Telemetry-based studies were conducted at 4 study sites within Minnesota's bear range (1981-2010): CNF (Chippewa National Forest, main study site in central bear range); VNP (Voyageurs National Park, northern fringe of range); Camp Ripley Military Reserve (near southern edge of range); NW (northwestern fringe of range).

.

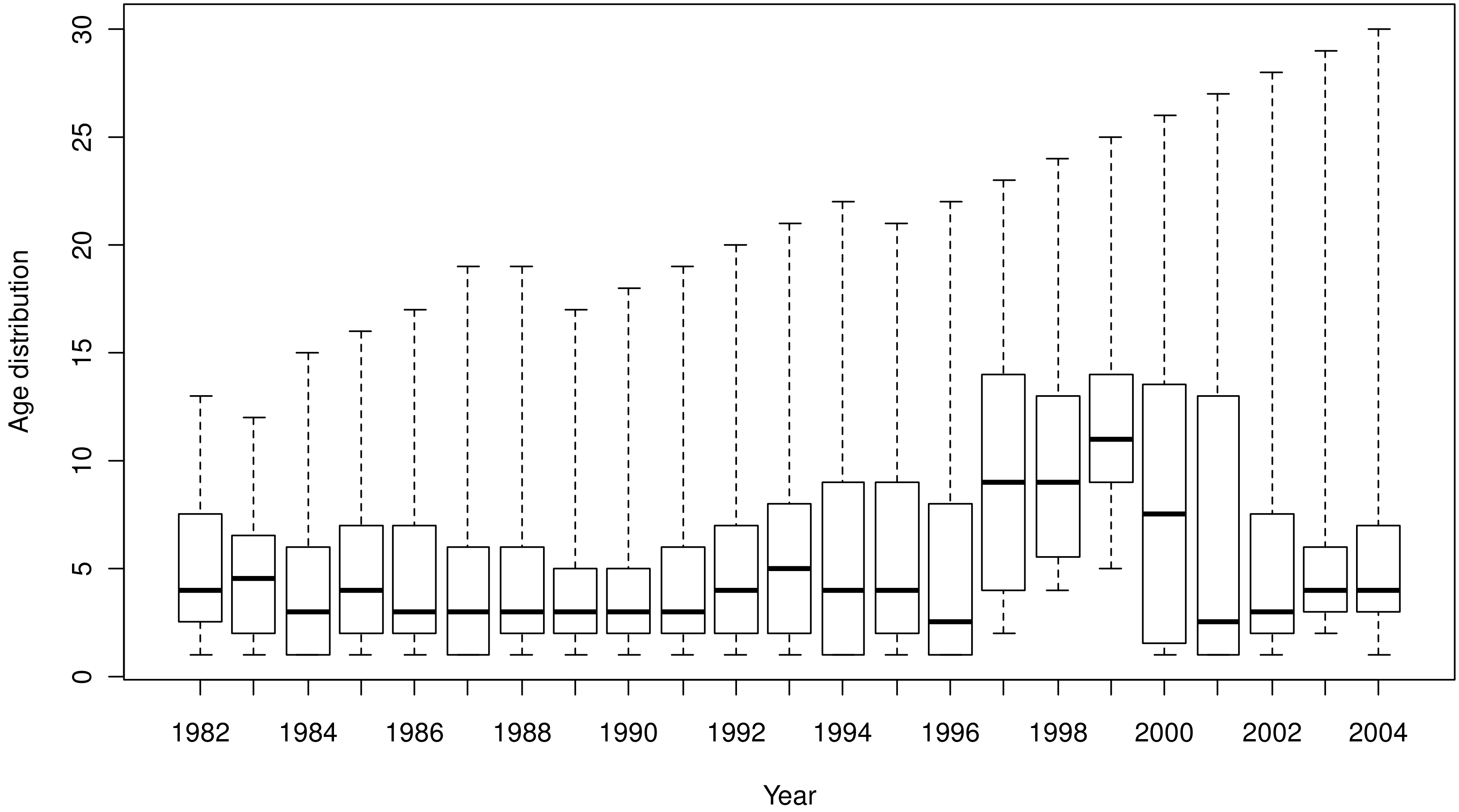

Figure S1.2. Age distribution of radio-collared bears from the Chippewa National Forest (CNF) study site. Boxes bound the 25th and 75th percentiles, solid line within the box indicates the median, and the whiskers extend to the range of the observations. The increasing age of the oldest study bear is due to one individual.
