# Supplementary material for: Integrated Population Modeling of Black Bears in Minnesota: Implications for Monitoring and Management
Source: PLoS One. 2010 Aug 12;5(8):e12114. doi: 10.1371/journal.pone.0012114 (PMC2920827; doi:10.1371/journal.pone.0012114)
Supplement: Appendix S2 — A description of the simulation study (methods) used to test the robustness of the integrated population modeling approach. (0.07 MB PDF) [file pone.0012114.s005.pdf]

Appendix S2: Simulation Study Methods

This supplementary appendix summarizes simulation methods used to test the robustness of the age-at-harvest modeling approach reported in Fieberg et al. "**Integrated Population Modeling of Black Bears in Minnesota: Implications for Monitoring and Management**."

We began by verifying that the AD model builder (ADMB) estimation program returned the correct parameter values when we used a deterministic simulation (i.e., operating) model (with fixed survival and harvest rates) to project population dynamics. We then tested our ADMB code using the eight simulation scenarios detailed below. In each case, we defined an operating model to describe true population and harvest dynamics, and we applied several estimation models. Estimation models had the same structure as the operating models, except where noted. We began by creating a *Baseline Scenario* to mimic (our best guess at) population dynamics of Minnesota (MN) black bears from 1980-2008. Six other scenarios were constructed by altering a single facet of the *Baseline Scenario*; a final scenario (the *Kitchen Sink Scenario*) was constructed by including all of these complications in the simulation model.

Operating Models

Parameters in the Baseline Scenario were determined from initial fits of age-at-harvest models to the MN black bear data (initial abundances, cub deviation parameters) or using data from a long-term (nearly 30 year) radio-telemetry study (harvest regression parameters, cub and adult survival rates; Appendix S1).

Baseline Scenario

- Initial (1980) abundance of males by age (cubs, 1, 2, …, 10+) = (1454, 439, 677, 293, 301, 112, 174, 90, 97, 18, 90)
- Initial (1980) abundance of females by age (cubs, 1, 2, …, 10+) = (1529, 599, 1274, 475, 483, 261, 431, 331, 191, 181, 408)
- Male survival probabilities (cubs, yearlings, age 2+) = (0.76, 0.88, 0.92)
- Female survival probabilities (cubs, yearlings, age 2+) = (0.88, 0.92, 0.97)
- Survival probabilities were constant over time.
- Proportion of cubs that were male = 0.5.
- Number of cubs in each year was generated by first multiplying female age-specific abundances by an age-specific reproduction function [number of cubs/female for ages 0-10+ = (0, 0, 0, 0.123, 0.703, 0.990, 1.088, 1.3, 1.3, 1.3, 1.3)]. This total number of cubs was then multiplied by a yearly deviation. Annual deviations in the 29 simulated years were set to (0.87, 1.08, 1.19, 1.15, 1.43, 0.85, 1.54, 1.26, 1.47, 0.98, 1.11, 1.43, 1.01, 1.49, 1.16, 1.06, 1.66, 0.97, 1.57, 1.26, 1.59, 1.31, 1.85, 1.28, 1.70, 1.69, 1.23, 1.33, 0.85). The values for annual deviations were chosen because they mimicked bear population trajectories estimated from initial fits of age-at-harvest models to MN black bear data. These deviations were kept constant across all simulations (within and among scenarios).
- Harvest rates were assumed to be a deterministic, nonlinear function of age (modeled using a natural cubic regression spline with 3 degrees of freedom), sex, food availability, and hunting effort, all assumed to be linear and additive on the log-log scale. Regression

parameters were set to values estimated from an analysis of radio telemetry data at a study site in the middle of the state of Minnesota (see Figure 2 in main text for a depiction of these relationships and Appendix S1 for a description of the methods used to analyze these data).

- Realized survival and harvest were determined using binomial random variables, to allow for demographic stochasticity.
- All harvested bears were assumed to be reported and aged correctly.

The next six scenarios are identical to the *Baseline Scenario*, except as noted below.

Scenario 2 (Stochastic Rates)

- Survival rates were modeled as stochastic, by adding a random normal deviate with mean = 0 and variance = 0.0625 to logit-transformed values from the *Baseline Scenario* (and then these rates were inverted; Figure S2.1)
- Harvest rates were modeled as stochastic, by adding a random normal deviate with mean = 0 and variance = 0.16 to complementary log-log-transformed values from the *Baseline Scenario* (and then these rates were inverted; Figure S2.2).

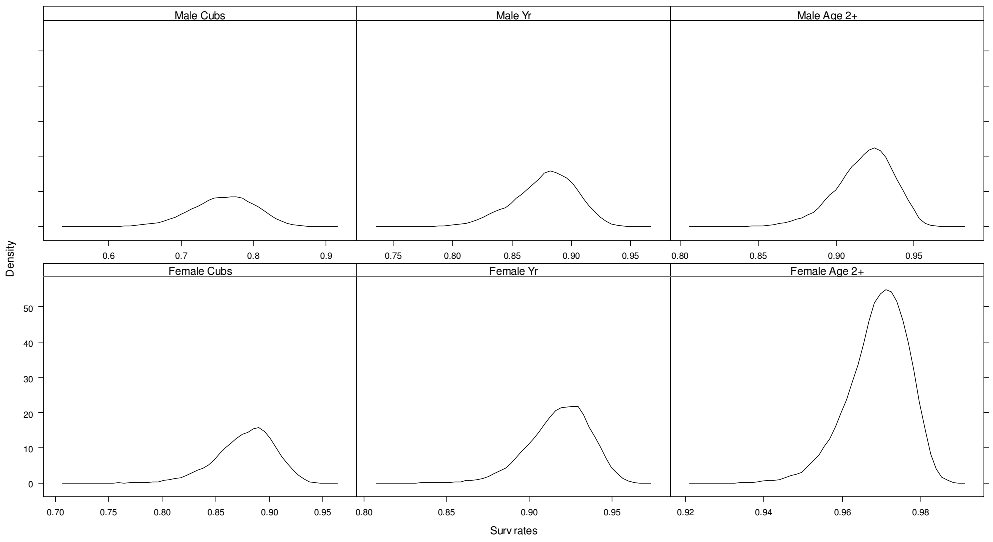

Figure S2.1. Probability distributions representing variability in non-hunting survival probabilities for male and female cubs, yearlings, and age 2+ bears in *Scenario 2* (*Stochastic Rates*). Distributions were estimated using 10,000 random draws from the stochastic model of survival in *Scenario 2* along with kernel density estimators with default smoothing parameters (constructed using the "density" function in R).

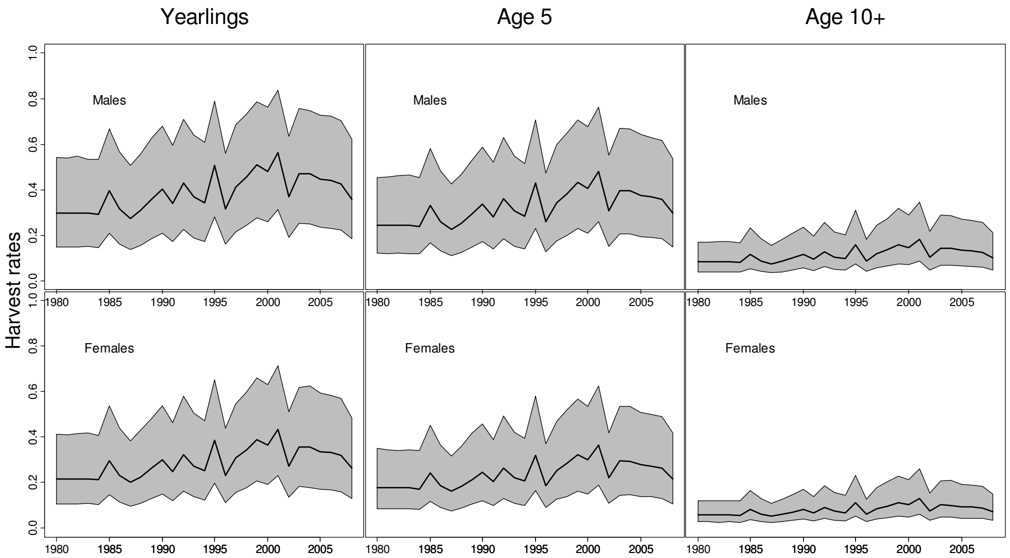

Figure S2.2. Variability in harvest rates for male and female yearlings, 5-year-old bears, and age 10+ bears using the model of stochastic harvest in *Scenario 2* (*Stochastic Rates*). Black lines give the harvest rates in the baseline scenario in which all variability is due to changes in natural food availability and hunting effort, whereas shaded areas encompass 95% of simulated values (from 10,000 random draws of the stochastic harvest model).

Scenario 3 (Trend in Harvest)

- A temporal trend in harvest rates (beyond that captured by natural food availability and hunter effort) was modeled by subtracting 0.02*(t-14) from the complementary log-log transformed deterministic harvest rate in year t given in the *Baseline Scenario* (Figure S2.3). This resulted in a decreasing trend in harvest rates (after accounting for the effect of food and hunting effort).

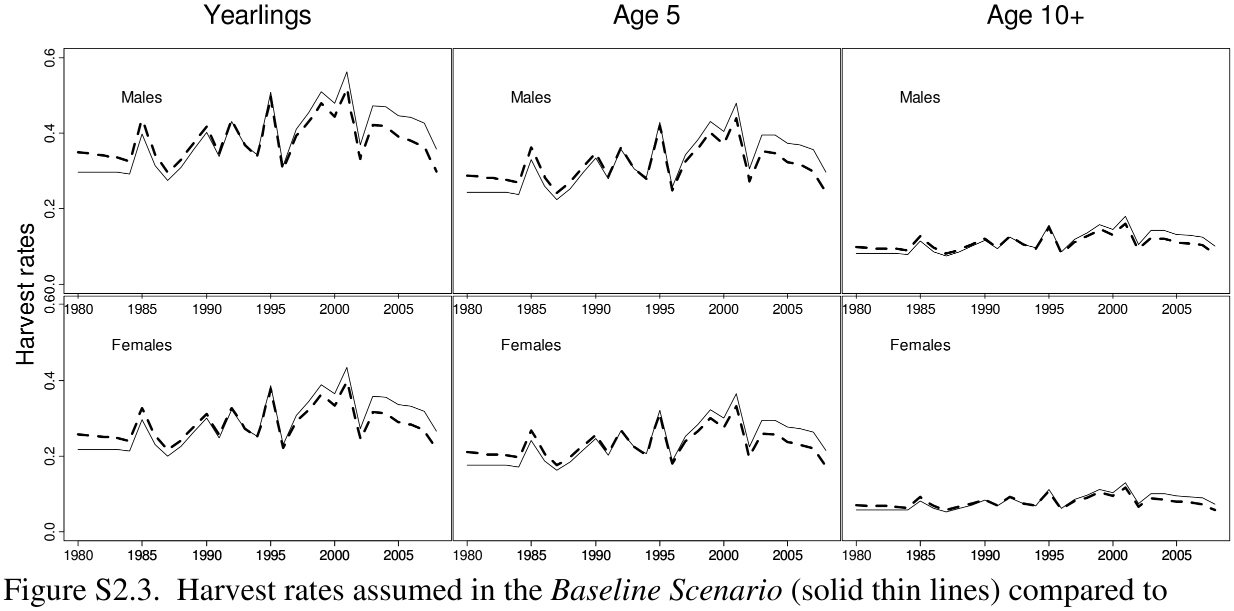

Figure S2.3. Harvest rates assumed in the *Baseline Scenario* (solid thin lines) compared to harvest rates assumed *in Scenario 3* (*Trend in Harvest*) (bold, dashed lines).

Scenario 4 (Incorrect Survival)

- Male survival rates of the operating model [(cubs, yearlings, age 2+) = (0.70, 0.84, 0.94)] differed from those of the estimation models (which were the same as in the *Baseline Scenario*).
- Female survival rates of the operating model [(cubs, yearlings, age 2+) = (0.80, 0.84, 0.97)] differed from those of the estimating models (which were the same as in the *Baseline Scenario*).

Scenario 5 (Reporting Error)

- Yearling bears were reported with probability = 0.5
- Age 2+ bears were reported with probability = 0.75
- Reported harvests were inflated by the overall reporting rate, assuming the age distribution in the reported harvest was the same as in the overall harvest.

Scenario 6 (Food x Sex Effect)

- We assumed the effect of food varied by sex. We estimated sex-specific effects by fitting a model with a food x sex interaction to telemetry data from the CNF study site (see Figure S2.4 and compare to Figure 2 in the main text, which depicts the **Baseline** *Scenario*; also, see Appendix S1 for a description of the methods used to analyze the telemetry data).

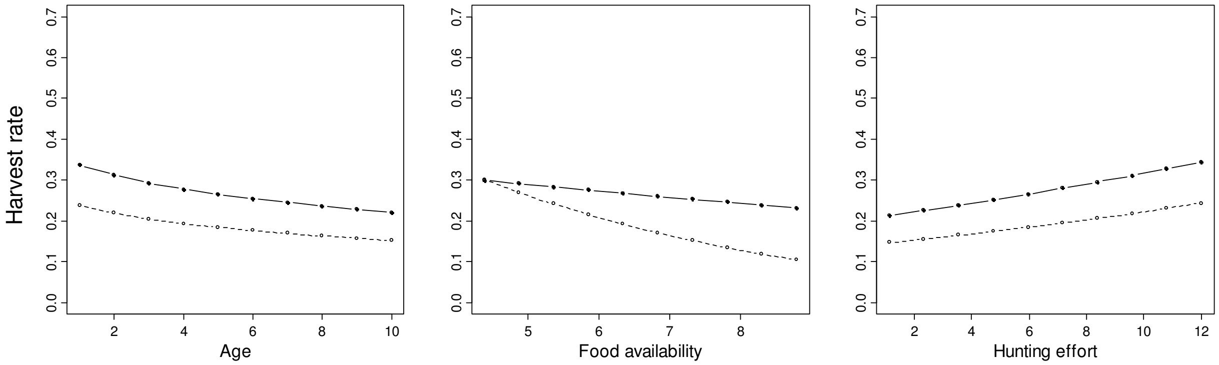

Figure S2.4. Harvest rates as a function of age, food availability, and license purchases for scenario 6 (Food x Sex Effect), separately for males (solid lines) and females (dotted lines). In each panel, covariates not displayed on the *x*-axis were held constant at values of age = 5, food availability = 6.5, and hunting effort 6.5.

Scenario 7 (Increasing S(t))

- A temporal trend in survival rates was modeled by adding 0.015*(*t*-14) from the logit transformed deterministic survival rates in the *Baseline Scenario* (Figure S2.5), and then these rates were inverted.

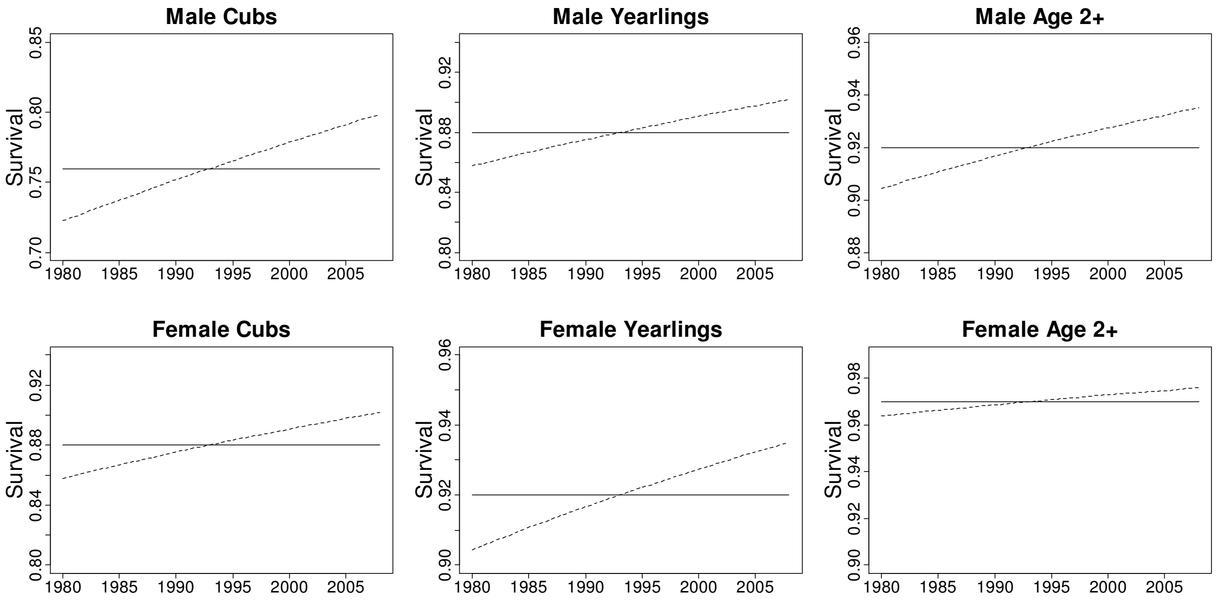

Figure S2.5. Trends in survival assumed in *Scenario 7* (*Increasing S*(*t*)) (dashed lines), compared to the *Baseline Scenario* (solid horizontal lines).

Scenario 8 (Kitchen Sink)

- Included all deviations noted in Scenarios 2-7.

Simulation of Mark-Recapture Data

Mark-recapture data were simulated in years 1991, 1997, and 2002 using normal random variables with means set equal to the true population size in those years (determined from the operating model) and with standard deviations set to give a CV of 8%.

Estimation Models

We ran 1000 simulations under each scenario, resulting in a total of 8,000 age-at-harvest and mark-recapture data sets. We fit all six estimation models (two harvest rate parameterizations x three penalty weights for the mark-recapture component of the objective function) to each data set to see how closely the estimation models could identify underlying parameter values in the simulation. This resulted in a total of 48,000 ADMB runs (although we only summarized the distribution of estimates in cases where the Hessian was positive definite, suggesting that a minimum had been obtained). A list of the estimated parameters for each of the harvest parameterizations is given in Box S1.
