# Supplementary material for: Integrated Population Modeling of Black Bears in Minnesota: Implications for Monitoring and Management
Source: PLoS One. 2010 Aug 12;5(8):e12114. doi: 10.1371/journal.pone.0012114 (PMC2920827; doi:10.1371/journal.pone.0012114)
Supplement: Appendix S3 — Additional table and figures summarizing the fits of integrated population models to Minnesota black bear data. (0.10 MB PDF) [file pone.0012114.s006.pdf]

Appendix S3: Application to MN Black Bear Data, Supporting Table and Figures

In this appendix, we include a supplemental table and several supplemental figures summarizing estimates from the 6 models (2 harvest rate parameterizations x 3 penalty weights for the mark-recapture component of the objective function) applied to Minnesota black bear data (1980-2008). Models are as described in Box S1.

Supplemental Table S3.1. Estimates of survival parameters from models fitted to MN black bear data (1980-2008).[1]

Model	Female yearling	Female adults	Male adults
H(*a, s, f, e; w*=0)	0.88	0.93	0.90
H(*a, s, f, e; w*=1)	0.88	0.94	0.92
H(*a, s, f, e; w*=200)	0.88	0.94	0.92
H(*a, s, yr; w*=0)	0.88	0.94	0.88
H(*a, s, yr; w*=1)	0.88	0.97	0.95
H(*a, s, yr; w*=200)	0.88	0.96	0.94

[1] Cub survival for males and females and male yearling survival were assumed known for all models and set equal to (0.76, 0.88, and 0.88) respectively.

List of Figures

Figure S3.1. Estimated harvest rates (averaged over ages) for years 1980-2008 for males (black circles, solid line) and females (open circles, dashed line).

Figure S3.2. Estimated harvest rates (averaged over years) as a function of age for males (black circles, solid line) and females (open circles, dashed line).

Figure S3.3. Estimated harvest rates as a function of food availability (top row) and hunting effort (bottom row), separately for males (black circles, solid line) and females (open circles, dashed line).

Figure S3.4. Observed total male harvests by year (points) and model predictions (solid lines).

Figure S3.5. Observed total female harvests by year (points) and model predictions (solid lines).

Figure S3.6. Estimated reproductive performance through time (i.e., number of cubs born/number of females age 5 or greater).

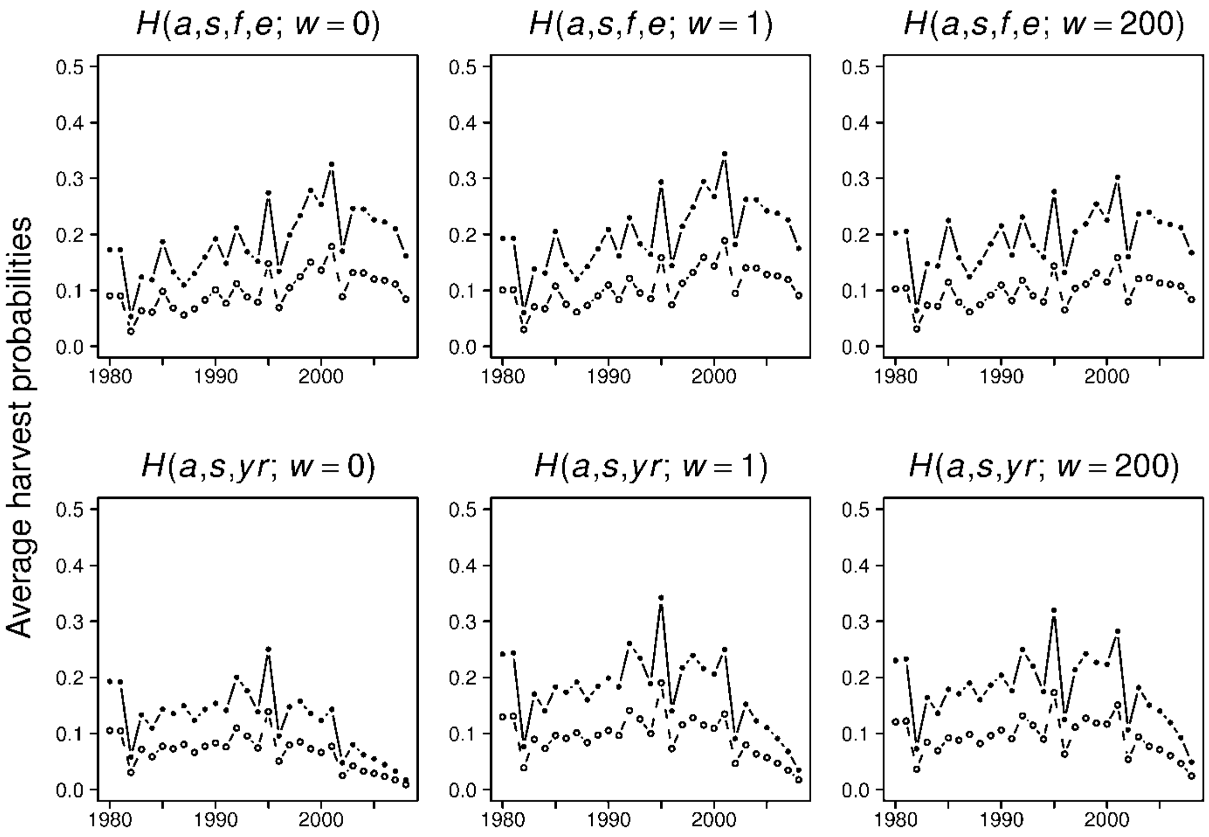

Figure S3.1. Estimated harvest rates (averaged over ages) for years 1980-2008 for males (black circles, solid line) and females (open circles, dashed line). *H*(*a, s, f, e*) estimators model temporal variability in harvest rates as a function of food availability and hunting effort indices, whereas the *H*(*a, s, yr*) estimators use an unstructured model for harvest rates. In both cases, *w* refers to the weight assigned to the mark-recapture component of the objective function used to fit the model.

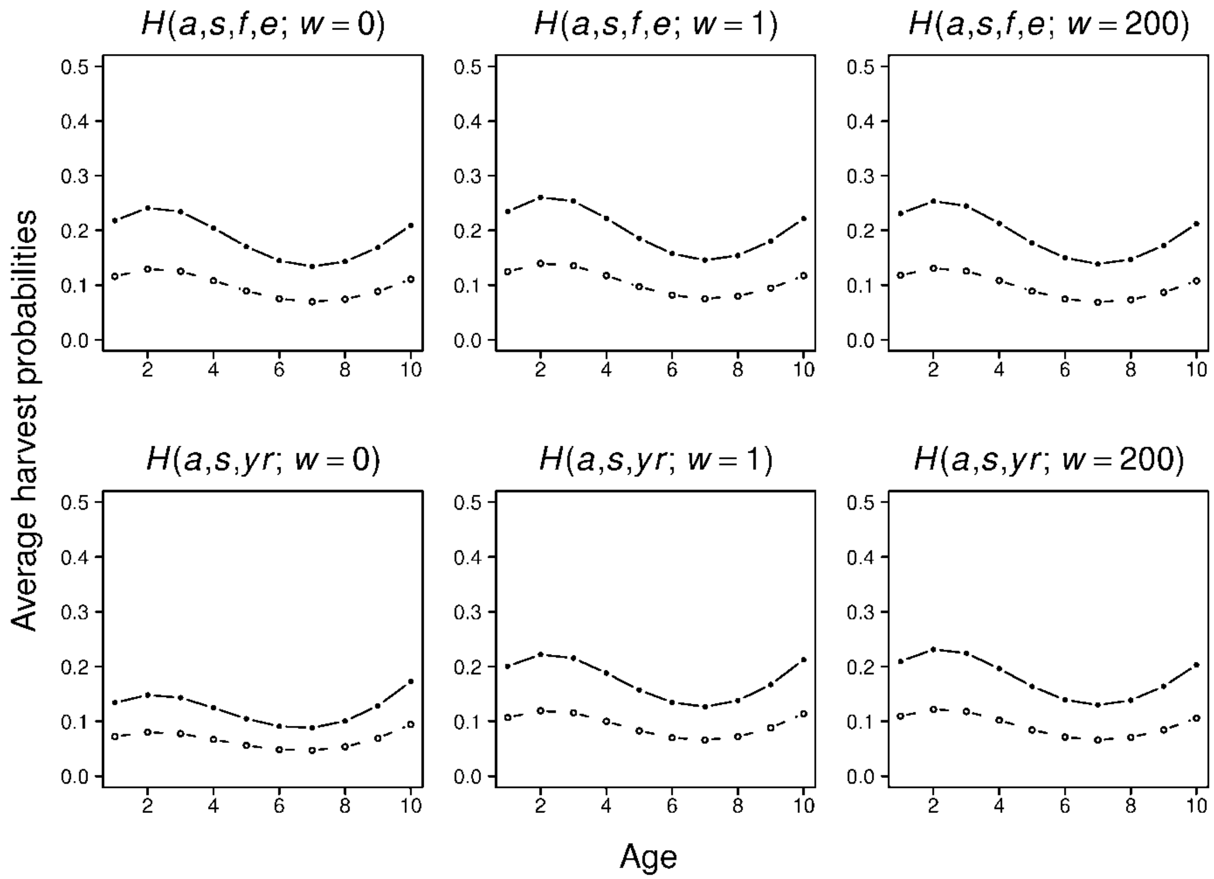

Figure S3.2. Estimated harvest rates (averaged over years) as a function of age for males (black circles, solid line) and females (open circles, dashed line). *H*(*a, s, f, e*) estimators model temporal variability in harvest rates as a function of food availability and hunting effort indices, whereas the *H*(*a, s, yr*) estimators use an unstructured model for harvest rates. In both cases, *w* refers to the weight assigned to the mark-recapture component of the objective function used to fit the model.

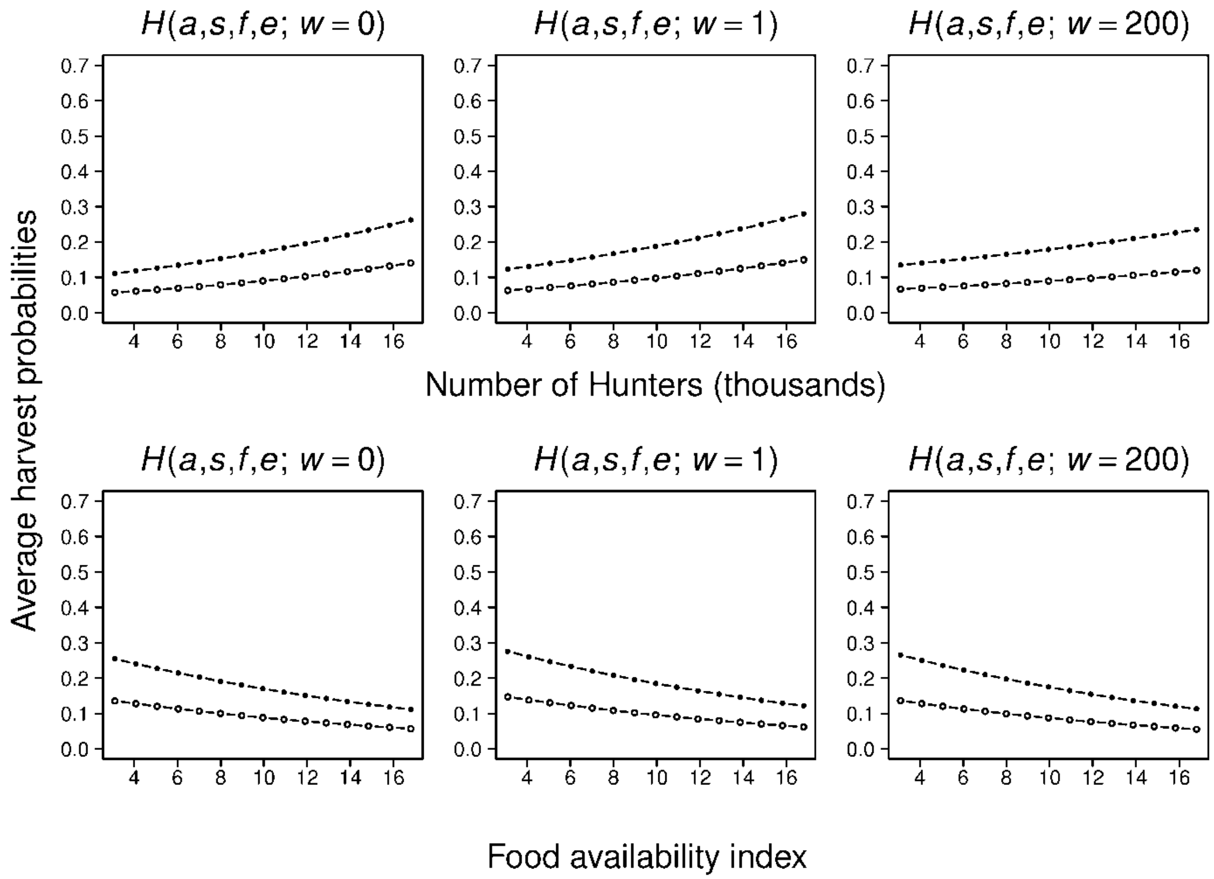

Figure S3.3. Estimated harvest rates as a function of food availability (top row) and hunting effort (bottom row), separately for males (black circles, solid line) and females (open circles, dashed line). *H*(*a, s, f, e*) estimators model temporal variability in harvest rates as a function of food availability and hunting effort indices, whereas the *H*(*a, s, yr*) estimators use an unstructured model for harvest rates. In both cases, *w* refers to the weight assigned to the mark-recapture component of the objective function used to fit the model.

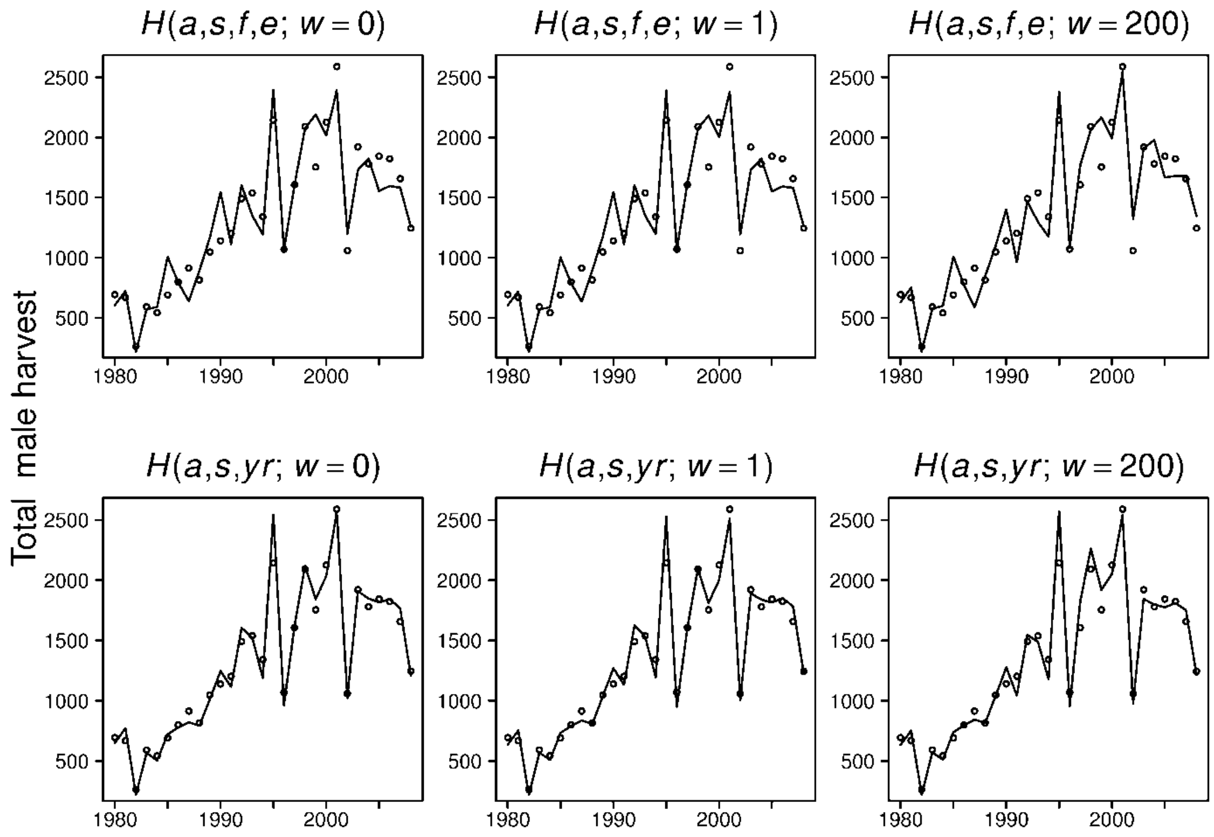

Figure S3.4. Observed total male harvest by year (points) and model predictions (solid lines). *H*(*a, s, f, e*) estimators model temporal variability in harvest rates as a function of food availability and hunting effort indices, whereas the *H*(*a, s, yr*) estimators use an unstructured model for harvest rates. In both cases, *w* refers to the weight assigned to the mark-recapture component of the objective function used to fit the model.

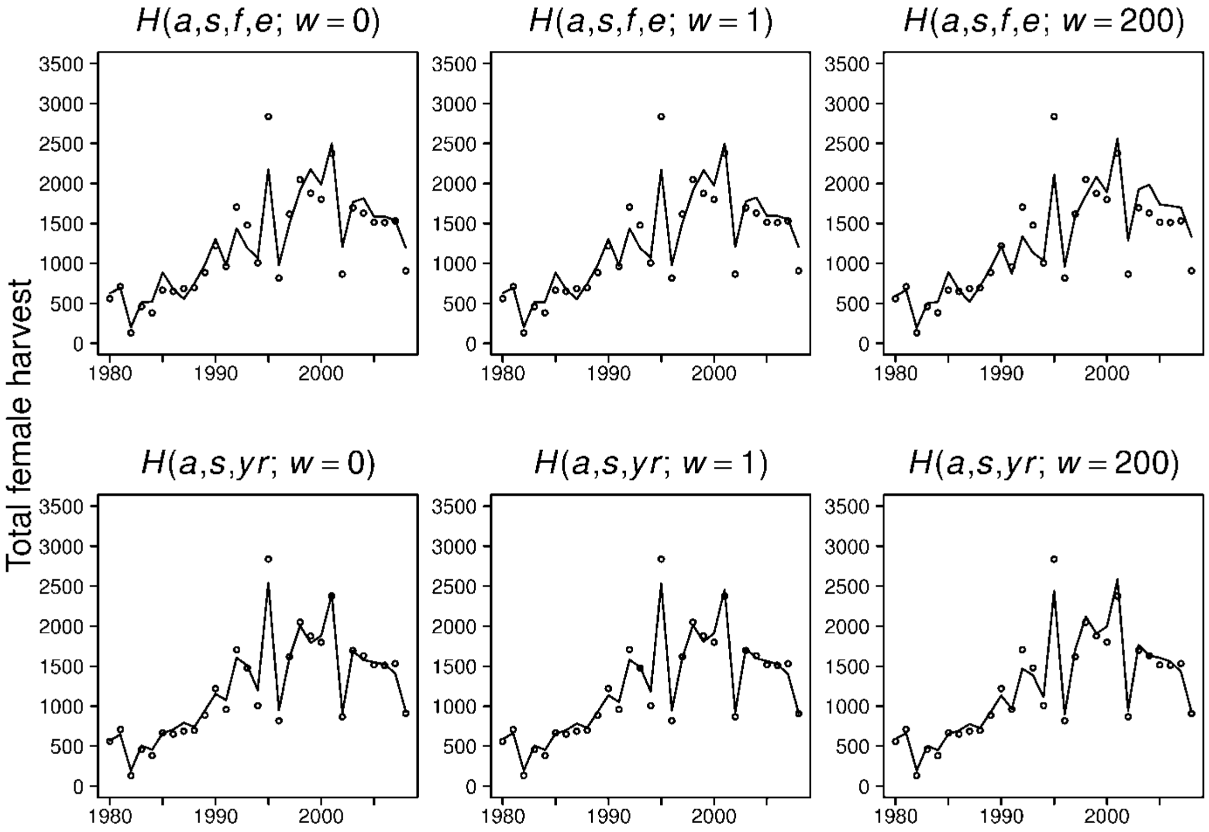

Figure S3.5. Observed total female harvest by year (points) and model predictions (solid lines). *H*(*a, s, f, e*) estimators model temporal variability in harvest rates as a function of food availability and hunting effort indices, whereas the *H*(*a, s, yr*) estimators use an unstructured model for harvest rates. In both cases, *w* refers to the weight assigned to the mark-recapture component of the objective function used to fit the model.

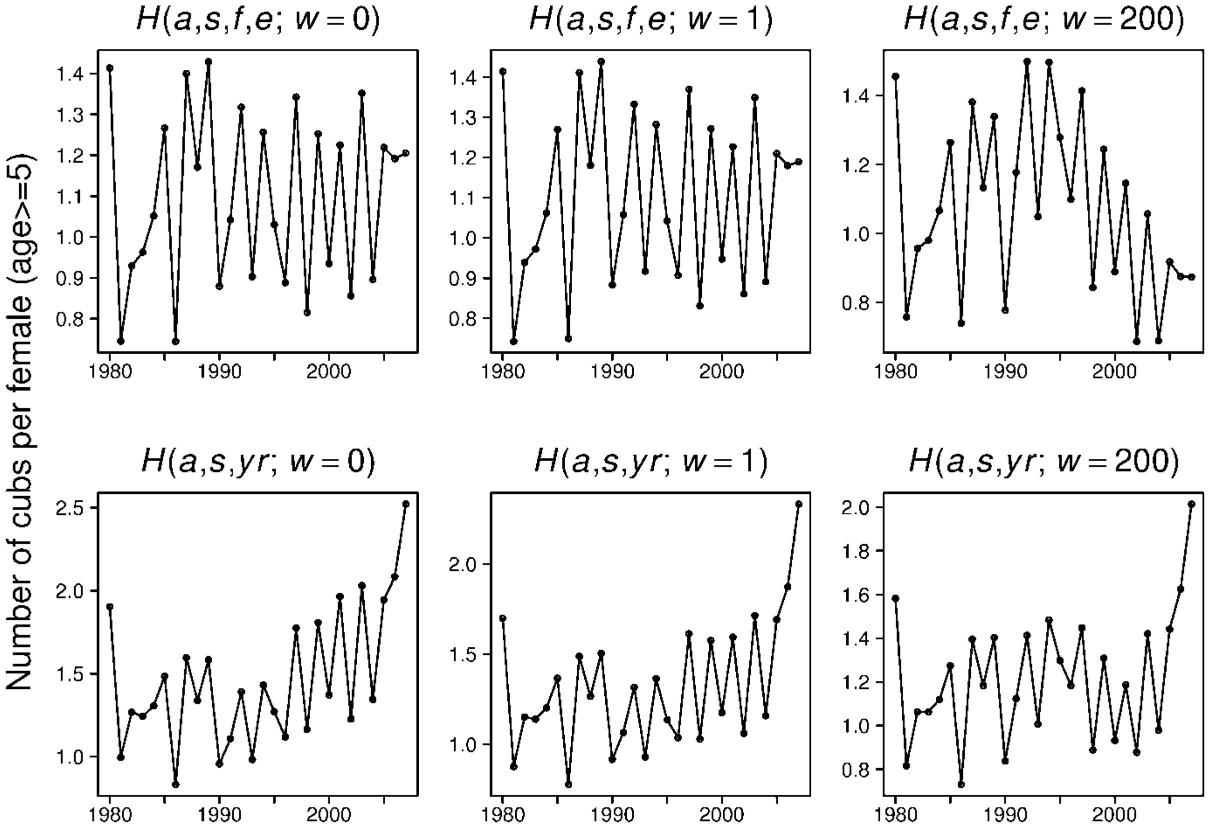

Figure S3.6. Estimated reproductive performance through time (i.e., number of cubs born/number of females age 5 or greater). *H*(*a, s, f, e*) estimators model temporal variability in harvest rates as a function of food availability and hunting effort indices, whereas the *H*(*a, s, yr*) estimators use an unstructured model for harvest rates. In both cases, *w* refers to the weight assigned to the mark-recapture component of the objective function used to fit the model.
